# Supplementary material for: Rapid and visual identification of β-lactamase subtypes for precision antibiotic therapy
Source: Nat Commun. 2024 Jan 24;15:719. doi: 10.1038/s41467-024-44984-y (PMC10808423; doi:10.1038/s41467-024-44984-y)
Supplement: Supplementary file 1 — Supplementary Information [file 41467_2024_44984_MOESM1_ESM.pdf]

Supporting Information for

Rapid and visual identification of *β*-lactamase subtypes for precision antibiotic therapy

Wenshuai Li[1, 2], Jingqi Li[1, 2], Hua Xu[3], Hongmei Gao[3], Dingbin Liu[1, 2,*]

[1] State Key Laboratory of Medicinal Chemical Biology, Frontiers Science Centers for Cell Responses and New Organic Matter, College of Chemistry, Nankai University, Tianjin 300071, China.

[2]Tianjin Key Laboratory of Molecular Recognition and Biosensing, Nankai University, Tianjin 300071, China.

[3]Department of Intensive Care Unit, Key Laboratory for Critical Care Medicine of the Ministry of Health, Emergency Medicine Research Institute, Tianjin First Center Hospital, School of Medicine, Nankai University, Tianjin 300071, China.

*Corresponding author: liudb@nankai.edu.cn

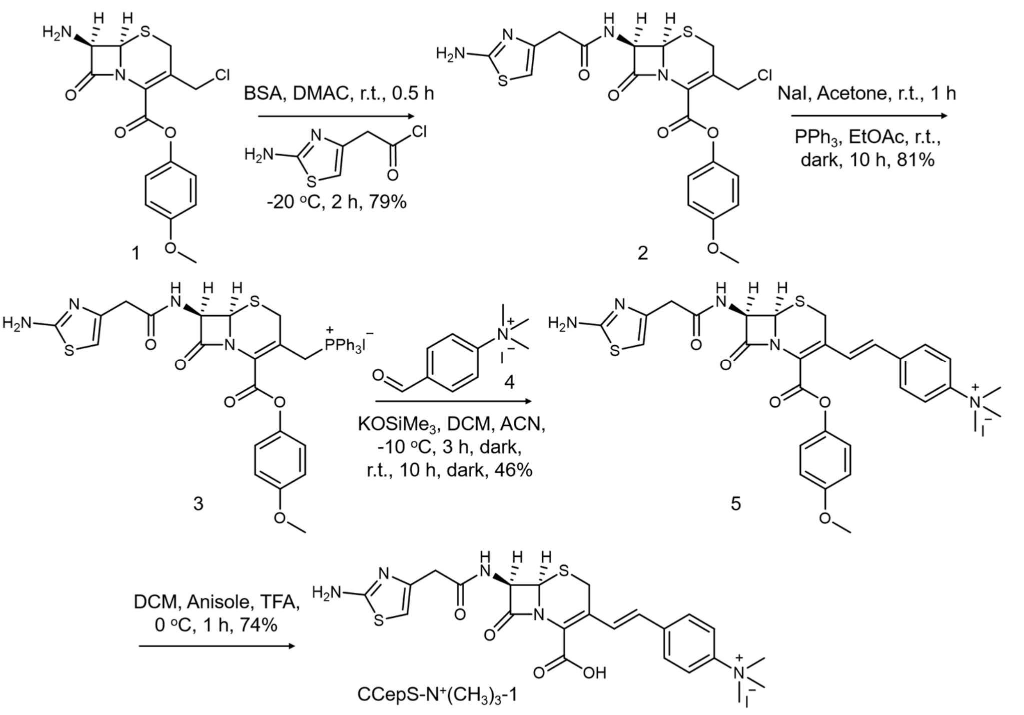

Supplementary Fig. 1 | Synthetic route of CCepS-N^{+}(CH$_3$)$_3$-1.

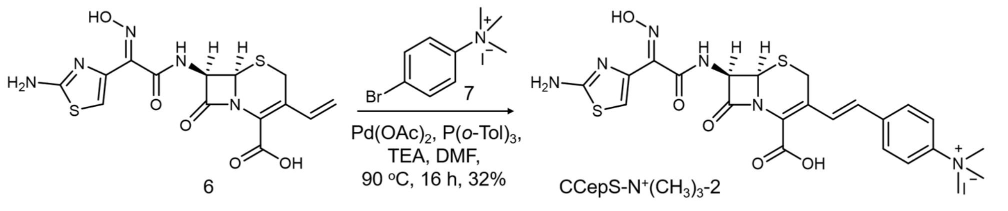

Supplementary Fig. 2 | Synthetic route of CCepS-N^{+}(CH$_3$)$_3$-2.

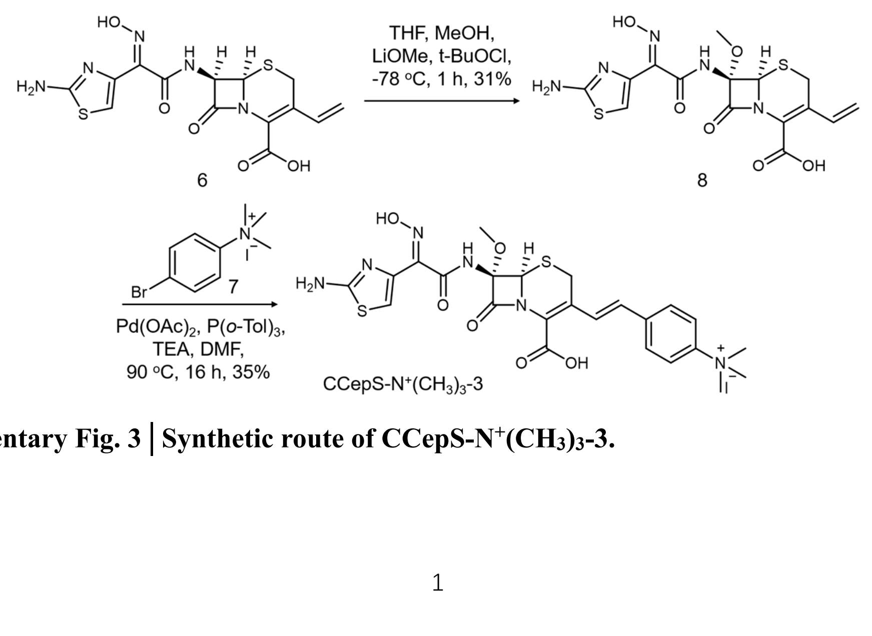

Supplementary Fig. 3 | Synthetic route of CCepS-N^{+}(CH$_3$)$_3$-3.

Compound 2

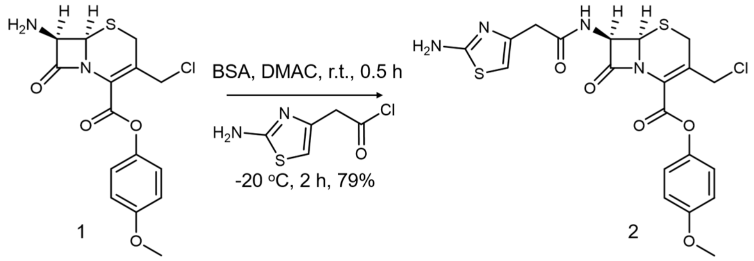

To a 250 mL boiling flask-3-neck was added compound 1 (3.54 g, 10 mmol) and bis-trimethylsilylacetamide (6.1 g, 30 mmol) in anhydrous *N,N*-dimethylacetamide (100 mL), and the mixture was stirred at room temperature for 0.5 h under Ar atmosphere. This mixture was cooled to -30 °C and 2-(2-aminothiazol-4-yl)acetyl chloride (3.52 g, 20 mmol) was added very slowly. The resulting reaction mixture was stirred at -20 °C for 2 h. When TLC indicated that the reaction was completed, the reaction solution was poured into ice water and extracted with EtOAc (100 mL), and washed with brine (150 mL×2) subsequently. The organic layer was dried over Mg_2SO_4 and concentrated. The crude product was purified by flash column chromatography with petroleum ether and ethyl acetate (PE: EA = 3: 1) as eluent to afford compound **2** (3.9 g, 79%) as an ashen-white solid. ^{1}H NMR (400 MHz, DMSO) δ 9.52 (d, J = 12 Hz, 1H), 7.37 (d, J = 8 Hz, 2H), 7.16 (s, 2H), 7.95 (d, J = 8 Hz, 2H), 7.16 (s, 1H), 5.28 (d, J = 8 Hz, 1H), 5.19 (d, J = 12 Hz, 1H), 4.60 (d, J = 8 Hz, 1H), 4.51 (d, J = 8 Hz, 1H), 3.85 (d, J = 16 Hz, 1H), 3.77 (d, J = 8 Hz, 3H), 3.74 (d, J = 16 Hz, 1H), 3.49 (d, J = 4 Hz, 1H), 3.47 (d, J = 4 Hz, 1H). ^{13}C NMR (101 MHz, DMSO) δ 170.80, 169.13, 161.63, 160.77, 158.86, 149.90, 130.91, 128.67, 127.25, 124.41, 114.29, 100.93, 69.66, 59.71, 55.62, 54.58, 43.78, 37.92, 27.08. HRMS (ESI) m/z calcd for $C_{20}H_{19}ClN_4O_5S_2^+$ [M-H]$^+$ 495.0485, found 495.0429.

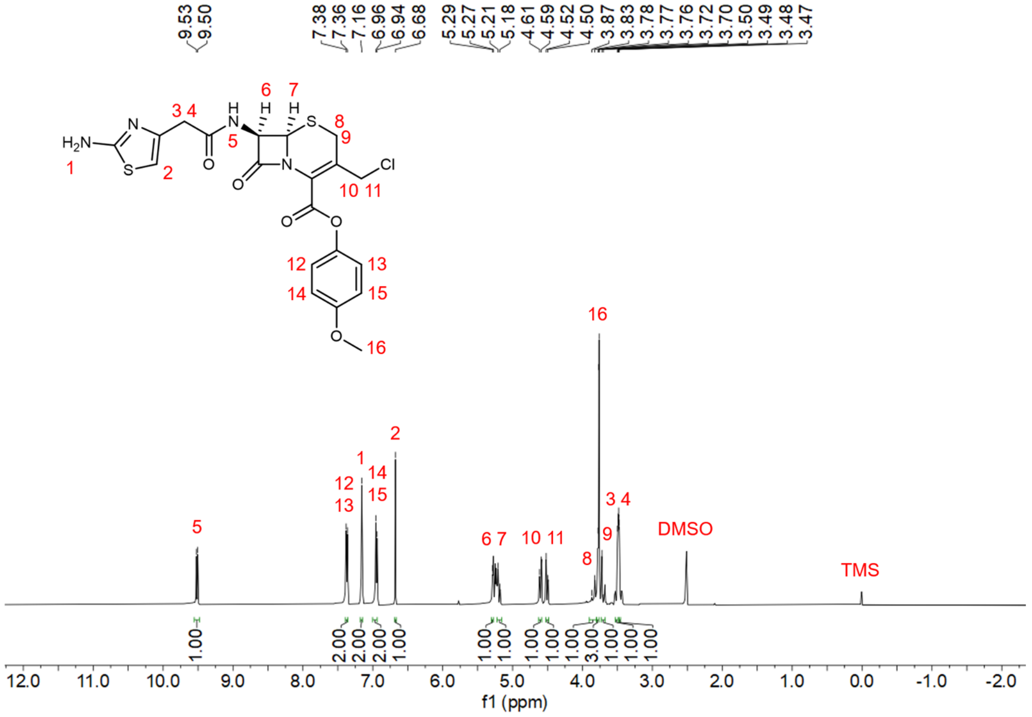

Supplementary Fig. 4 | ^{1}H NMR spectrum of compound 2 in DMSO.

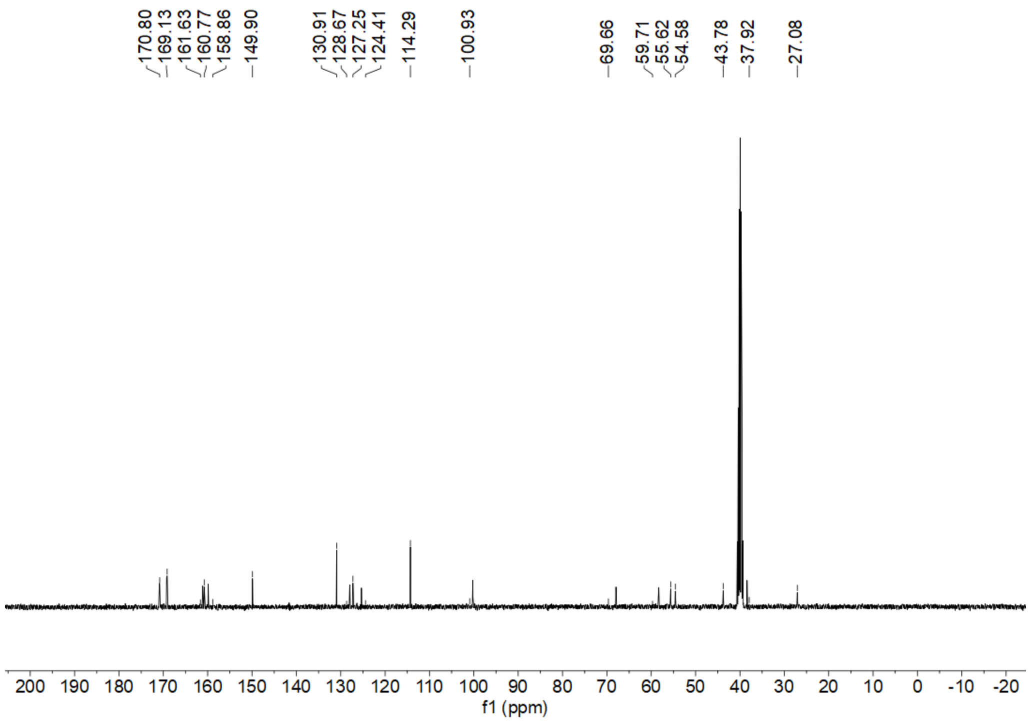

Supplementary Fig. 5 | ^{13}C NMR spectrum of compound 2 in DMSO.

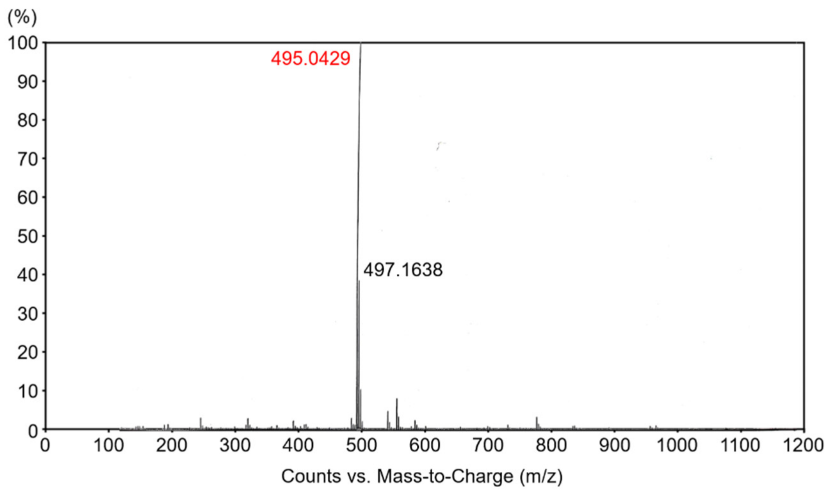

Supplementary Fig. 6 | HRMS spectrum (ESI) of compound 2.

Compound 3

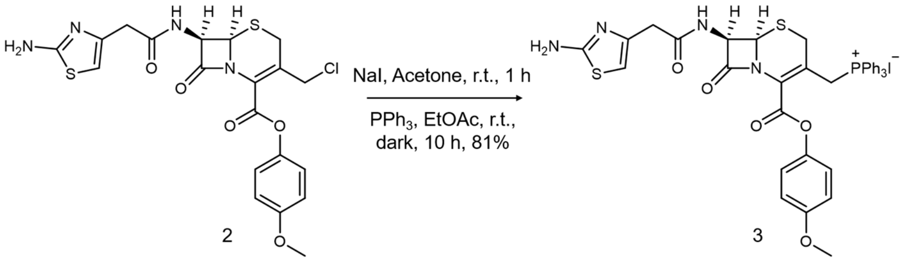

To a 250 mL boiling flask-3-neck was added compound **2** (2.47 g, 5 mmol) and sodium iodide (0.75 g, 25 mmol) in anhydrous acetone (120 mL), and the mixture was stirred at room temperature for 1 h under Ar atmosphere. The solvent was removed on rotary evaporator and the residue was dissolved in EtOAc (80 mL) and washed with sodium thiosulfate (50 mL), water (50 mL×2), and brine (50 mL×2). The organic phase was dried (Na_2SO_4) and the solvent was removed by rotary evaporator to reduce the volume to approximately 30 mL. Triphenylphosphine (2.62 g, 10 mmol) was added and the mixture was stirred at room temperature in the dark for 10 h under Ar atmosphere. A light brown solid (compound **3**) was formed which was filtered and washed with hexane (3.44 g, 81%). ^{1}H NMR (400 MHz, DMSO) δ 9.52 (d, *J* = 12 Hz, 1H), 7.81-7.55 (m, 15H), 7.32 (d, *J* = 12 Hz, 1H), 7.16 (d, *J* = 8 Hz, 2H), 6.93 (d, *J* = 12 Hz, 2H), 6.68 (s, 1H), 5.29 (d, *J* = 4 Hz, 1H), 5.22 (d, *J* = 8 Hz, 1H), 4.60 (d, *J* = 16 Hz, 1H), 4.52 (d, *J* = 4 Hz, 1H), 3.85 (d, *J* = 16 Hz, 1H), 3.77 (d, *J* = 8 Hz, 3H), 3.69 (d, *J* = 8 Hz, 1H), 3.36 (d, *J* = 12 Hz, 1H), 3.31 (d, *J* = 12 Hz, 1H). ^{13}C NMR (101 MHz, DMSO) δ 171.37, 169.11, 162.92, 160.77, 159.88, 150.69, 134.33, 130.91, 130.16, 127.96, 127.25, 126.04, 125.33, 124.48, 121.10, 118.80, 118.00, 114.29, 108.06, 67.95, 58.86, 56.09, 53.79, 44.26, 36.88, 28.25. HRMS (ESI) m/z calcd for $C_{38}H_{34}N_4O_5PS_2^+$ $[M-I]^+$ 721.1703, found 721.1735.

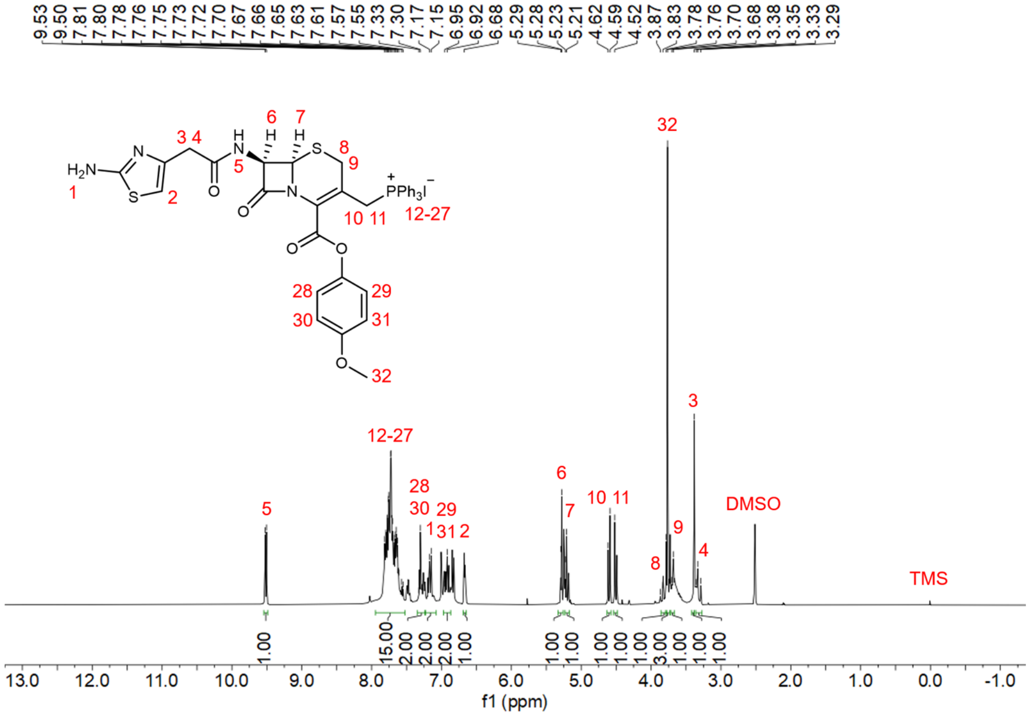

Supplementary Fig. 7 | ^{1}H NMR spectrum of compound 3 in DMSO.

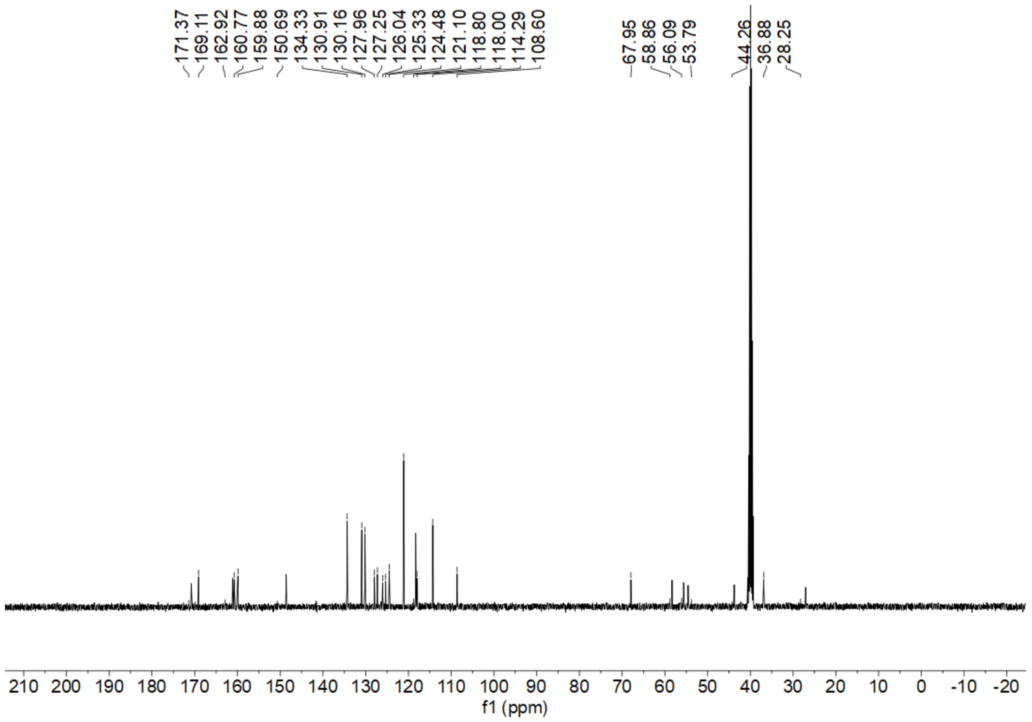

Supplementary Fig. 8 | ^{13}C NMR spectrum of compound 3 in DMSO.

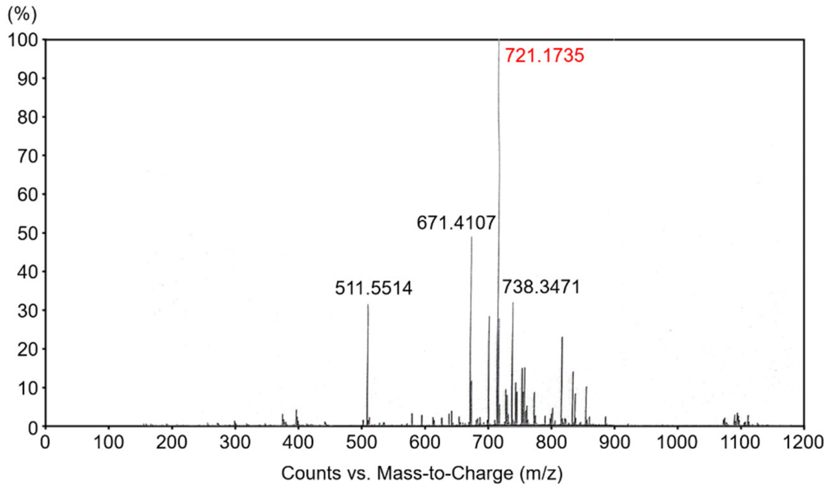

Supplementary Fig. 9 | HRMS spectrum (ESI) of compound 3.

Compound 4

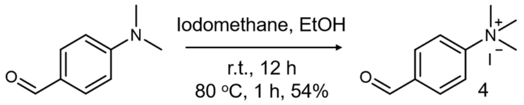

To a 150 mL boiling flask-3-neck was added 4-Dimethylaminobenzaldehyde (0.75 g, 5 mmol), iodomethane (2.13 g, 15 mmol), and anhydrous ethanol (50 mL) under Ar atmosphere. The resulting reaction mixture was stirred at 25 °C for 12 h and refluxed for 1 h until the formation of yellowish solids. After removing the solids, the solution was recrystallized at -20 °C to yield precipitates, which were washed with water and ether to obtain white solid compound **4** (0.79 g, 54%). Characterization data of the compound: ^{1}H NMR (400 MHz, $CDCl_3$) δ 9.68 (s, H), 7.67 (d, J = 8 Hz, 2H), 6.62 (d, J = 8 Hz, 2H), 3.00 (s, 9H). ^{13}C NMR (101 MHz, $CDCl_3$) δ 190.44, 154.30, 133.10, 124.70, 110.96, 39.37. HRMS (ESI) m/z calcd for $C_{10}H_{14}NO^+$ $[M-I]^+$ 164.1070, found 164.1039.

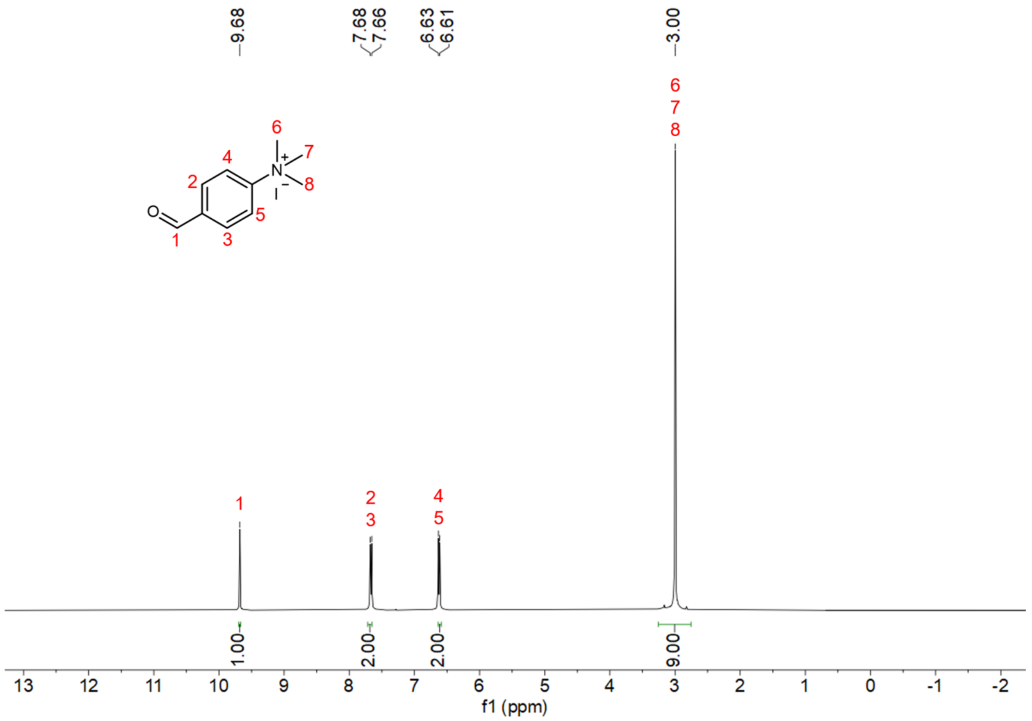

Supplementary Fig. 10 | ^{1}H NMR spectrum of compound 4 in $CDCl_3$.

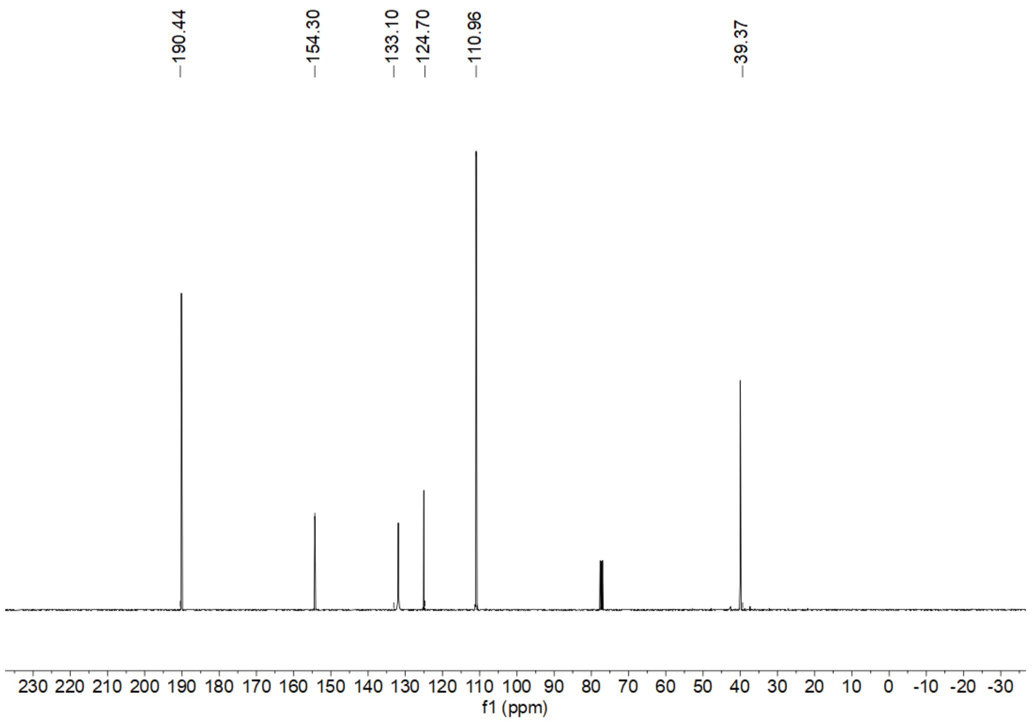

Supplementary Fig. 11 | ^{13}C NMR spectrum of compound 4 in $CDCl_3$.

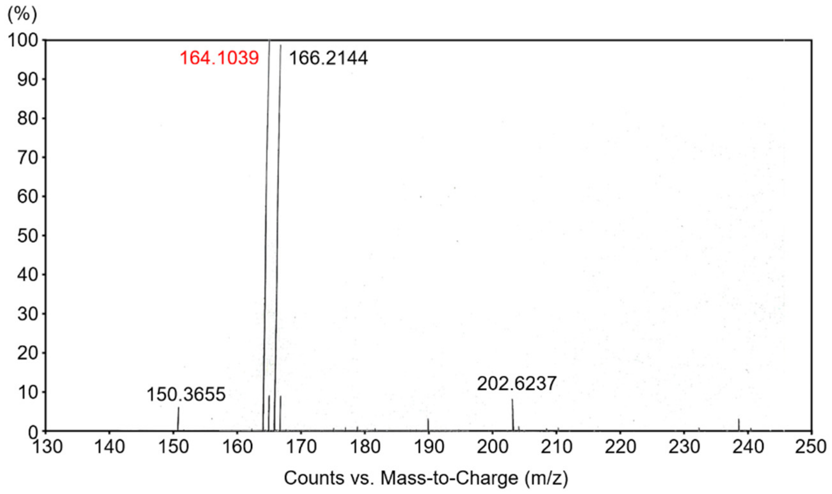

Supplementary Fig. 12 | HRMS spectrum (ESI) of compound 4.

Compound 5

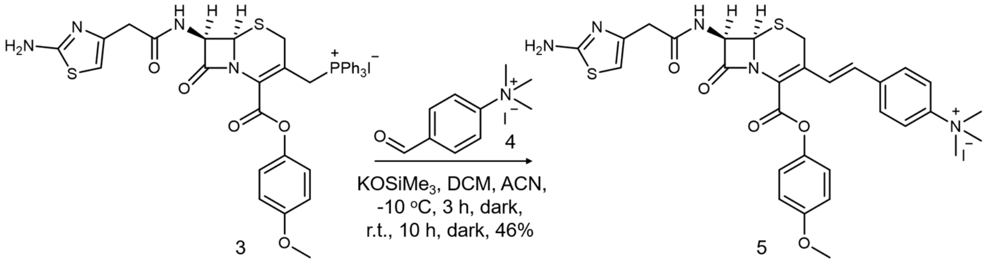

To a 150 mL boiling flask-3-neck was added compound **3** (1.7 g, 2 mmol) in anhydrous CH_2Cl_2 (30 mL), and potassium trimethylsilanolate (280 mg, 2 mmol) in acetonitrile (10 mL) was added to this solution in the dark at -10 °C, then the mixture was stirred at room temperature for 1 h under Ar atmosphere. A solution of compound **4** (2.33 g, 8 mmol) in CH_2Cl_2 (10 mL) was added and the solution was stirred in the dark at -10 °C for another 2 h and at room temperature for 10 h. When TLC indicated that the reaction was completed, the reaction solution was diluted with CH_2Cl_2 (200 mL) and washed with brine (150 mL×2) subsequently. The organic layer was dried over Mg_2SO_4 and concentrated. The crude product was purified by flash column chromatography with petroleum ether and ethyl acetate (PE: EA =3: 1) as eluent to afford compound **5** (0.67 g, 46%) as a tan solid. ^{1}H NMR (400 MHz, DMSO) δ 9.54 (d, J = 8 Hz, 1H), 7.52 (d, J = 8 Hz, 2H), 7.44 (d, J = 8 Hz, 2H), 7.25 (s, 2H), 7.18 (d, J = 8 Hz, 1H), 7.11 (d, J = 8 Hz, 2H), 7.02 (d, J = 8 Hz, 1H), 6.77 (d, J = 4 Hz, 1H), 6.71 (d, J = 12 Hz, 1H), 5.30 (d, J = 8 Hz, 1H), 5.17 (d, J = 8 Hz, 1H), 3.95 (d, J = 12 Hz, 1H), 3.77 (d, J = 4 Hz, 9H), 3.68 (d, J = 8 Hz, 1H), 3.6-3.51 (m, 3H), 3.34 (d, J = 4 Hz, 1H), 3.32 (d, J = 4 Hz, 1H). ^{13}C NMR (101 MHz, DMSO) δ 171.00, 170.67, 161.11, 160.77, 159.88, 151.39, 149.13, 130.91, 129.43, 127.96, 127.53, 127.25, 124.81, 119.28, 114.29, 108.70, 67.95, 58.46, 58.31, 55.62, 54.58, 43.78, 37.57, 27.08. HRMS (ESI) m/z calcd for $C_{30}H_{32}N_5O_5S_2^+$ $[M-I]^+$ 606.1840, found 606.1953.

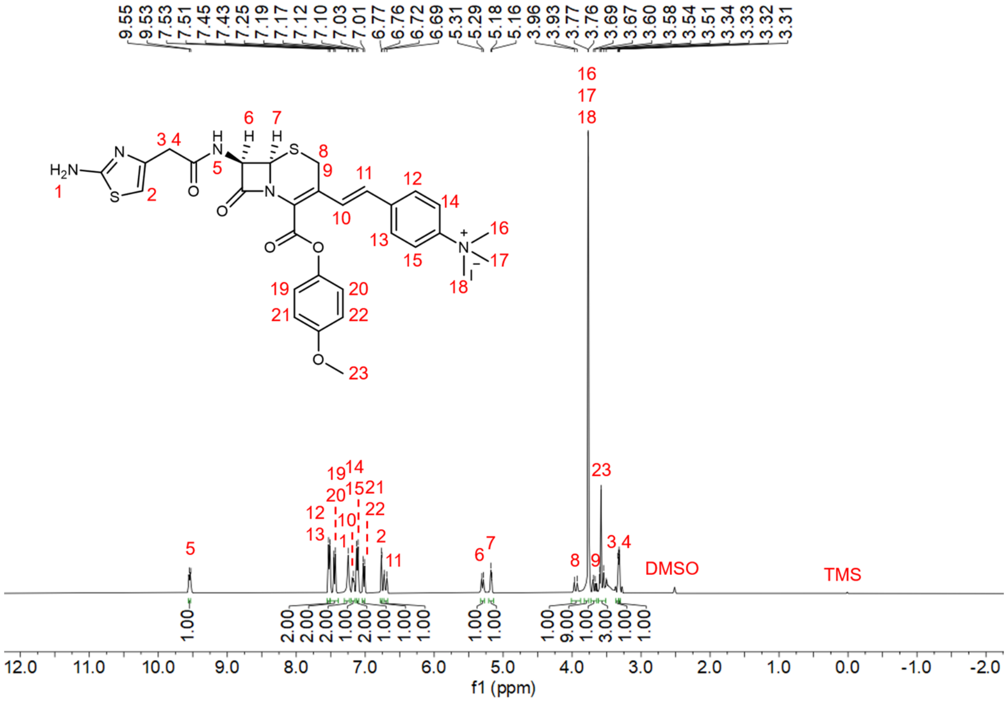

Supplementary Fig. 13 │ ^{1}H NMR spectrum of compound 5 in DMSO.

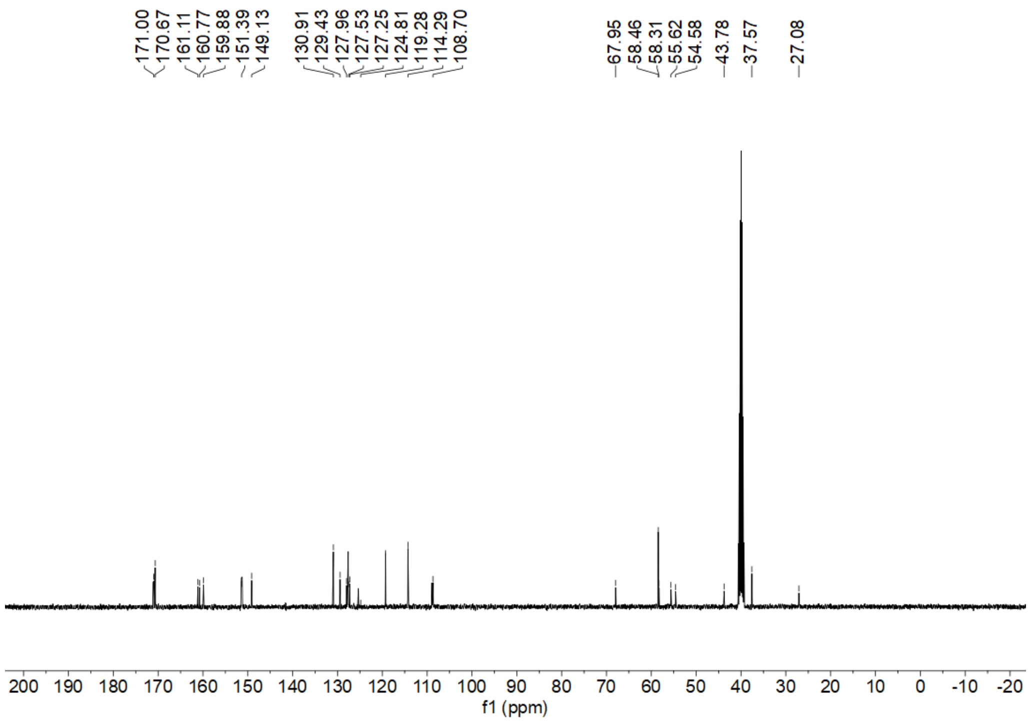

Supplementary Fig. 14 | ^{13}C NMR spectrum of compound 5 in DMSO.

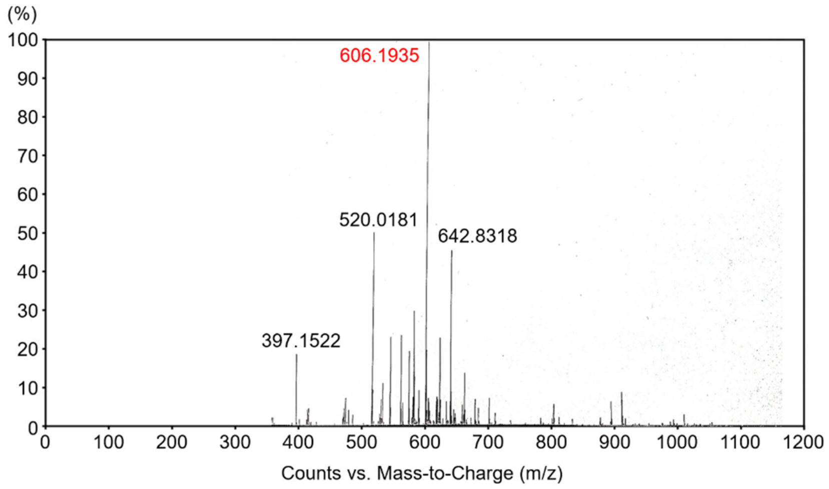

Supplementary Fig. 15 | HRMS spectrum (ESI) of compound 5.

CCepS-N$^+$(CH$_3$)$_3$-1

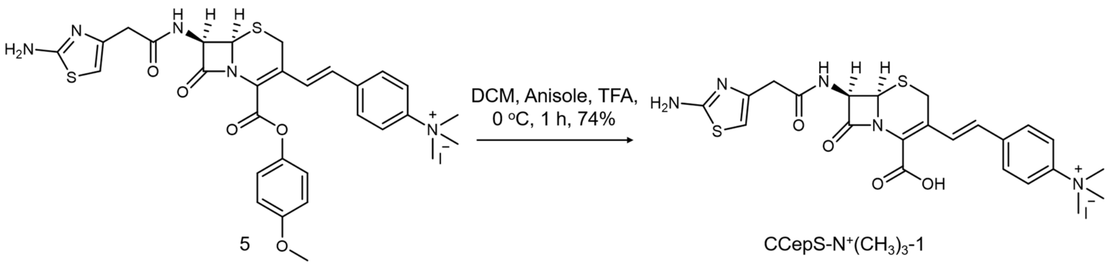

To a 150 mL boiling flask-3-neck was added compound **5** (0.73 g, 1 mmol), TFA (5 mL), anisole (0.5 mL), and anhydrous CH_2Cl_2 (50 mL) under Ar atmosphere. The resulting reaction mixture was stirred in the dark at 0 °C for 1 h. When TLC indicated that the reaction was completed, the reaction solution was diluted with CH_2Cl_2 (100 mL) and washed with brine (150 mL×2) subsequently. The organic layer was dried over Mg_2SO_4 and concentrated. The crude product was purified by flash column chromatography with dichloromethane and methanol (DCM: MeOH = 10: 1) as eluent to afford **CCepS-N$^+$(CH$_3$)$_3$-1** (0.46 g, 73%) as a tan solid. ^{1}H NMR (400 MHz, DMSO) δ 9.52 (d, J = 8 Hz, 1H), 7.33 (d, J = 8 Hz, 2H), 7.17 (s, 2H), 7.00 (d, J = 8 Hz, 1H), 6.91 (d, J = 8 Hz, 2H), 6.62 (d, J = 12 Hz, 1H), 6.57 (d, J = 8 Hz, 1H), 5.33 (d, J = 12 Hz, 1H), 5.20 (d, J = 8 Hz, 1H), 3.85 (d, J = 16 Hz, 1H), 3.75 (d, J = 4 Hz, 9H), 3.61 (d, J = 8 Hz, 1H), 3.56 (d, J = 8 Hz, 1H), 3.50 (d, J = 8 Hz, 1H). ^{13}C NMR (101 MHz, DMSO) δ 168.69, 164.43, 163.74, 144.08, 141.05, 132.47, 130.14, 128.04, 125.98, 124.72, 119.80, 117.73, 107.26, 59.22, 58.35, 52.94, 23.69. HRMS (ESI) m/z calcd for $C_{23}H_{26}N_5O_4S_2^+$ [M-I]$^+$ 500.1421, found 500.1427.

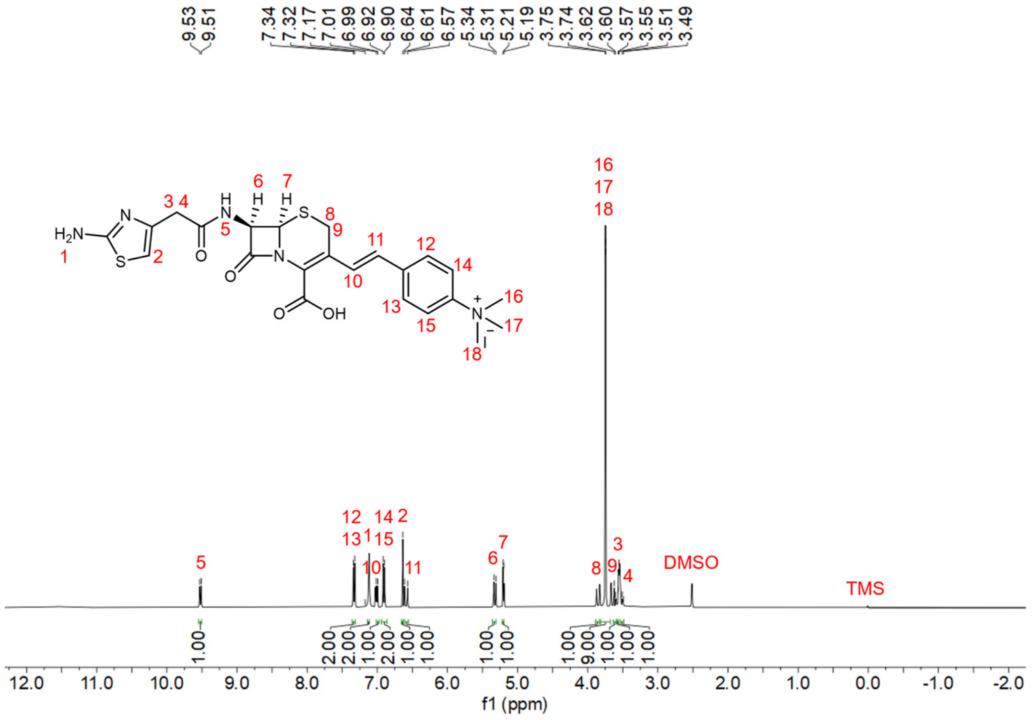

Supplementary Fig. 16 | ^{1}H NMR spectrum of CCepS-N$^+$(CH$_3$)$_3$-1 in DMSO.

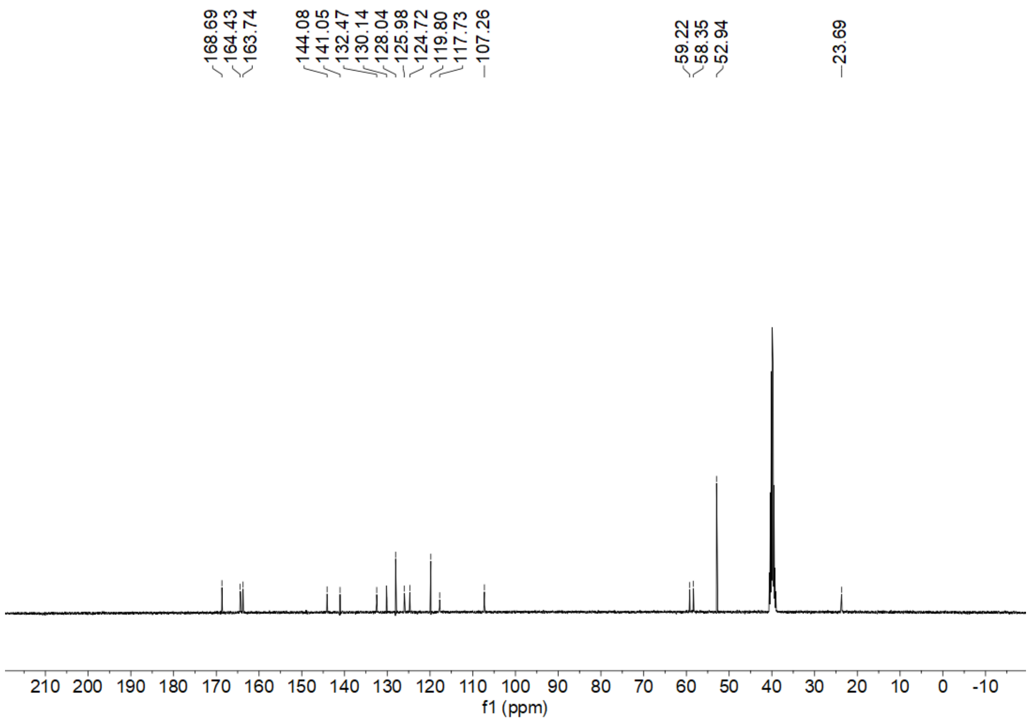

Supplementary Fig. 17 | ^{13}C NMR spectrum of CCepS-N$^+$(CH$_3$)$_3$-1 in DMSO.

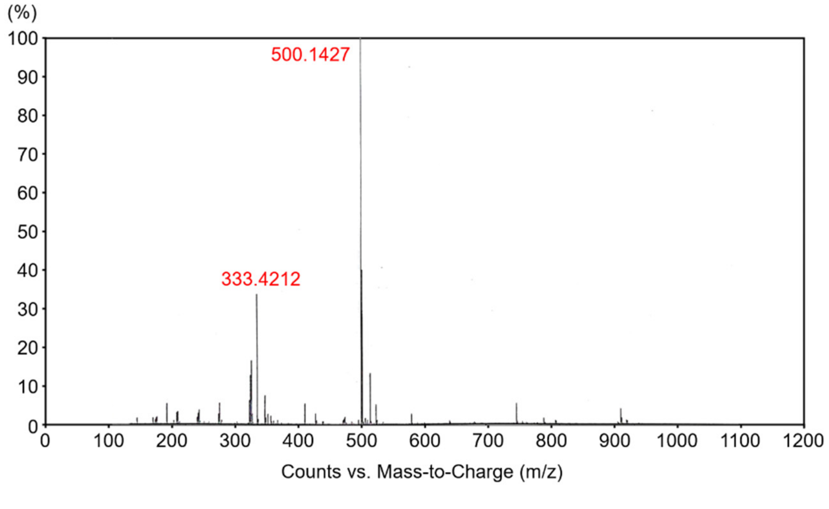

Supplementary Fig. 18 | HRMS spectrum (ESI) of CCepS-N$^+$(CH$_3$)$_3$-1.

CCepS-N$^+$(CH$_3$)$_3$-2

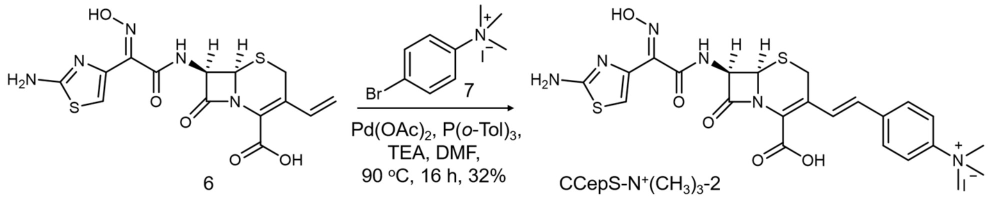

To a 150 mL boiling flask-3-neck was added compound **6** (1.98 g, 5 mmol), compound **7** (5.11 g, 15 mmol), Pd(OAc)$_2$ (673.53 mg, 3 mmol), P(o-Tol)$_3$ (913.11 mg, 3 mmol), triethylamine (3 mL) and anhydrous N, N-dimethylformamide (50 mL) under Ar atmosphere. The resulting reaction mixture was stirred at 90°C for 16 h. When TLC indicated that the reaction was completed, the reaction solution was diluted with ethyl acetate (30 mL) and washed with 0.2 M HCl (30 mL), NaHCO$_3$ saturated aqueous solution (30 mL) and brine (50 mL) sequentially. The organic layer was dried over Mg$_2$SO$_4$ and concentrated. The crude product was purified by flash column chromatography with dichloromethane and methanol (DCM: MeOH = 10: 1) as eluent to afford **CCepS-N$^+$(CH$_3$)$_3$-2** (1.38 g, 42%) as a tan solid. ^{1}H NMR (400 MHz, DMSO) δ 11.34 (s, 1H), 9.52 (d, J = 8 Hz, 1H), 7.39 (d, J = 8 Hz, 2H),7.16 (s, 2H), 7.06 (d, J = 12 Hz, 1H),6.94 (d, J = 8 Hz, 2H), 6.68 (s, 1H), 6.62 (d, J = 16 Hz, 1H), 5.32 (d, J = 12 Hz, 1H), 5.20 (d, J = 8 Hz, 1H), 3.85 (d, J = 16 Hz, 1H), 3.70 (s, 9H), 3.57 (d, J = 12 Hz, 1H). ^{13}C NMR (101 MHz, DMSO) δ 168.17, 163.82, 163.23, 148.51, 143.60, 141.72, 131.97, 127.41, 125.47, 124.23, 120.10, 117.23, 106.72, 58.72, 57.85, 54.48, 23.18. HRMS (ESI) m/z calcd for $C_{23}H_{25}N_6O_5S_2^+$ [M-I]$^+$ 529.1323, found 529.1332.

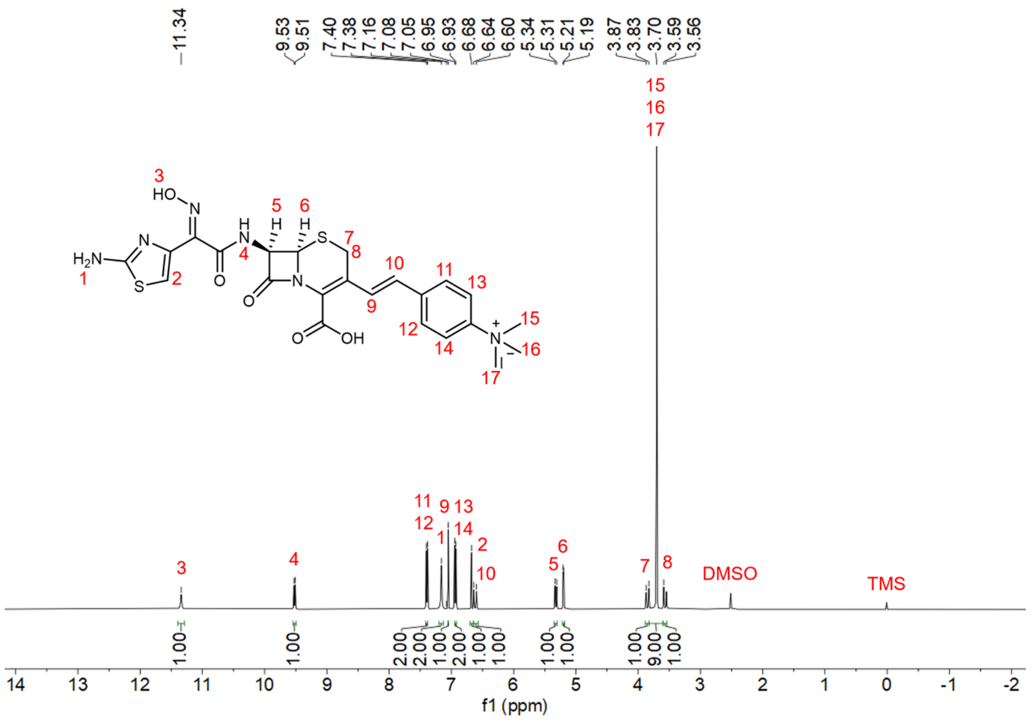

Supplementary Fig. 19 | ^{1}H NMR spectrum of CCepS-N$^+$(CH$_3$)$_3$-2 in DMSO.

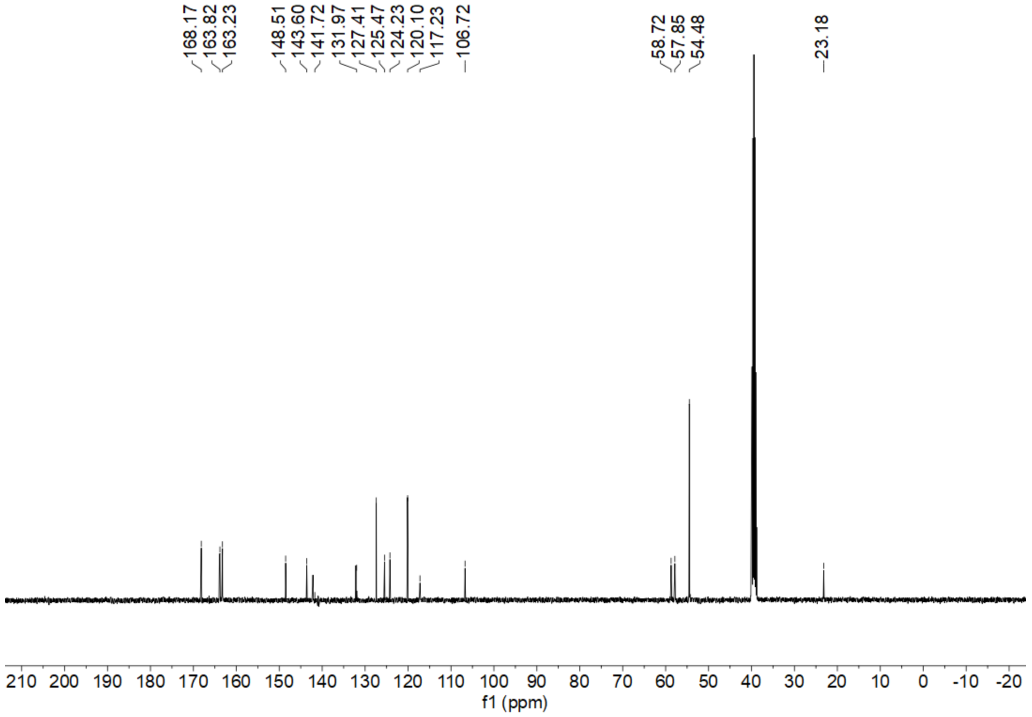

Supplementary Fig. 20 | ^{13}C NMR spectrum of CCepS-$N^+(CH_3)_3$-2 in DMSO.

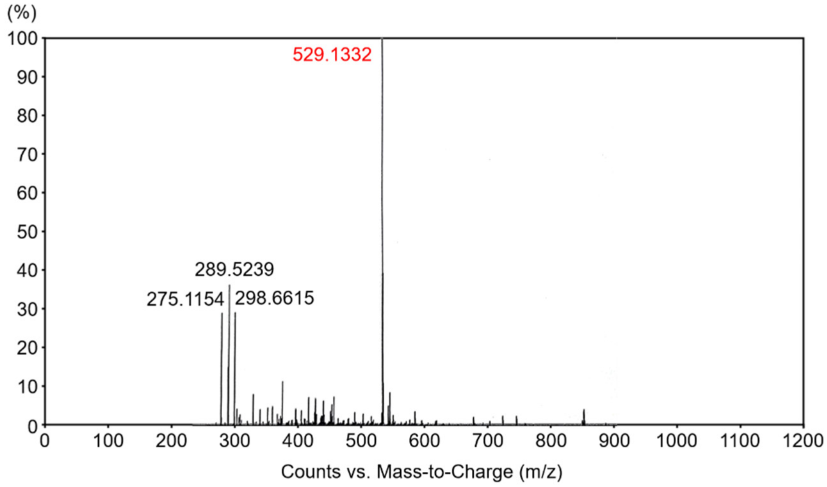

Supplementary Fig. 21 | HRMS spectrum (ESI) of CCepS-$N^+(CH_3)_3$-2.

Compound 8

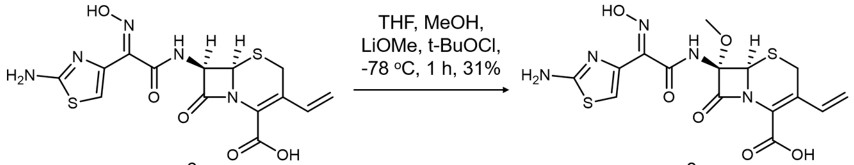

6 8

To a 150 mL boiling flask-3-neck was added compound **6** (1.98 g, 5 mmol) in anhydrous THF (50 mL), and LiOMe (753.42 mg, 20 mmol) in THF (3 mL) and MeOH (8 mL) was added to this solution at -78 °C under Ar atmosphere. After 10 min t-butyl hypochlorite (1 mL, 8.35 mmol) was added and after a further 1 h the mixture was poured into iced water (100 mL) containing NH_4Cl and $Na_2S_2O_5$ then extracted with EtOAc (80 mL×2). The organic layer was dried over Mg_2SO_4 and concentrated. The crude product was purified by flash column chromatography with dichloromethane and methanol (DCM: MeOH = 10: 1) as eluent to afford compound **8** (0.66 g, 31%) as an ashen-white solid. ^{1}H NMR (400 MHz, DMSO) δ 13.69 (s, 1H), 11.35 (s, 1H), 9.68 (d, J = 8 Hz, 1H), 7.17 (s, 2H), 6.95-6.88 (m, 1H), 6.68 (s, 1H), 5.82 (d, J = 4 Hz, 1H), 5.79 (d, J = 4 Hz, 1H), 5.60 (d, J = 16 Hz, 1H), 5.20 (d, J = 12 Hz, 1H), 3.85 (d, J = 16 Hz, 1H), 3.58 (d, J = 8 Hz, 1H), 3.37-3.28 (m, 3H). ^{13}C NMR (101 MHz, DMSO) δ 168.69, 164.31, 163.74, 149.00, 144.08, 132.47, 125.98, 124.72, 117.37, 107.26, 59.22, 58.35, 49.56, 23.68. HRMS (ESI) m/z calcd for $C_{15}H_{15}N_5O_6S_2^+$ [M-H]$^+$ 426.0464, found 426.0933.

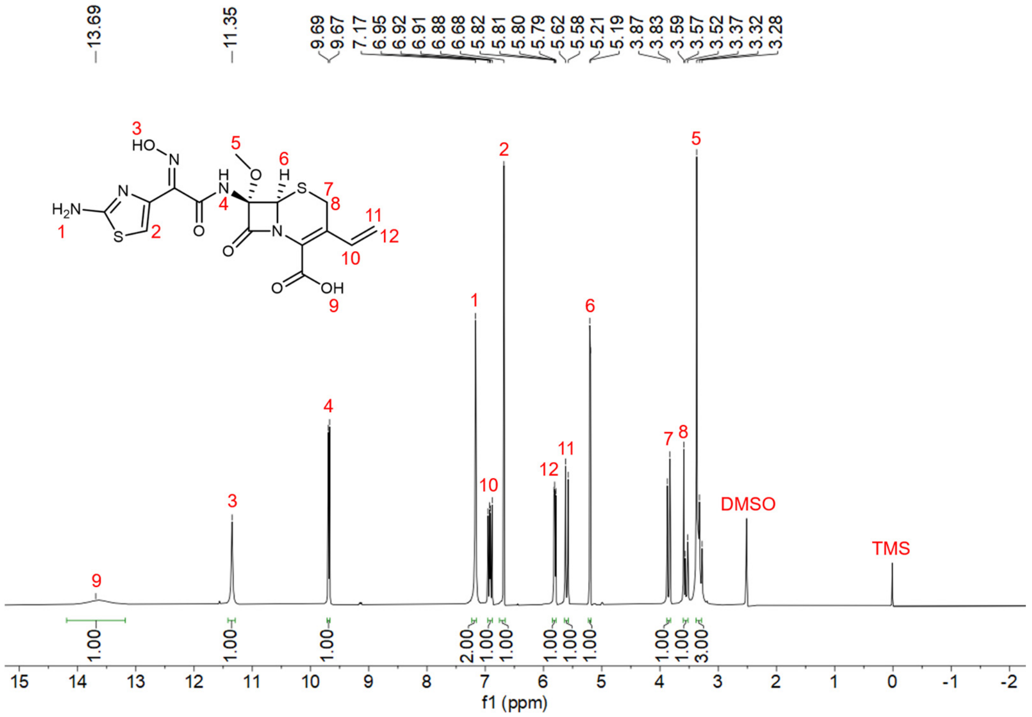

Supplementary Fig. 22 | ^{1}H NMR spectrum of compound 8 in DMSO.

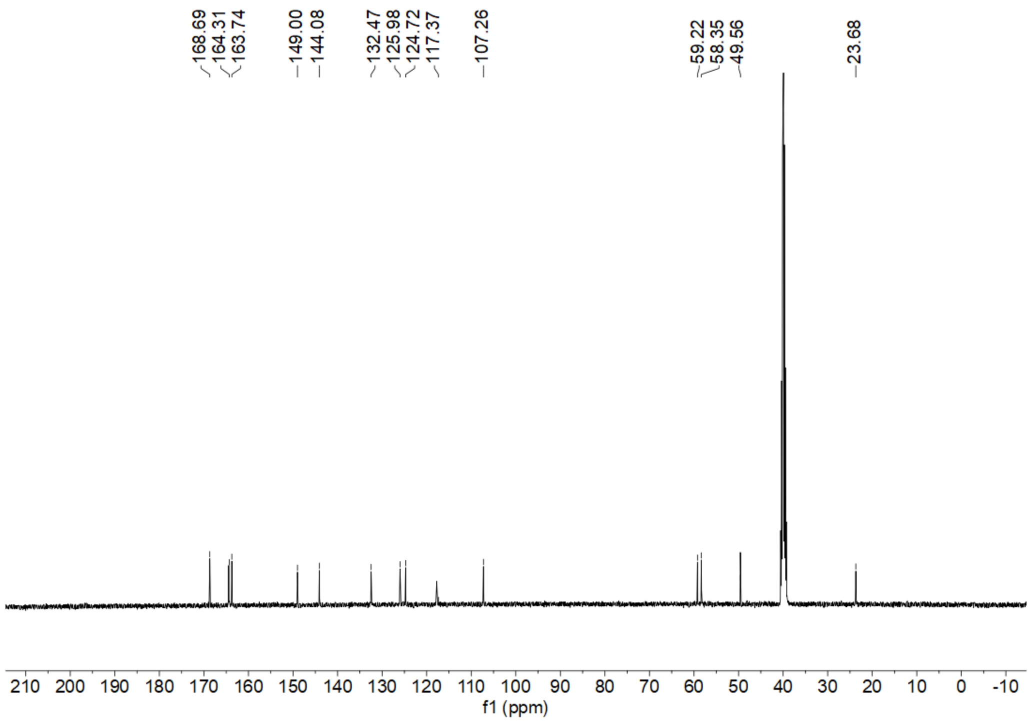

Supplementary Fig. 23 | ^{13}C NMR spectrum of compound 8 in DMSO.

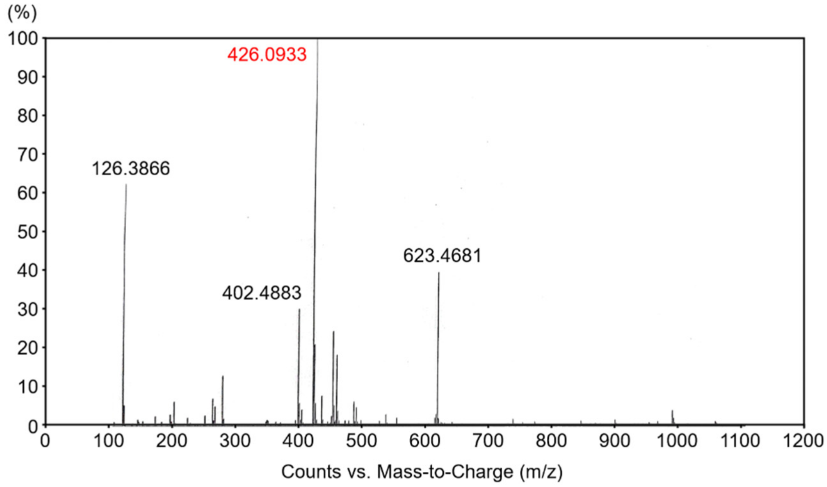

Supplementary Fig. 24 | HRMS spectrum (ESI) of compound 8.

CCepS-N+(CH3)3-3

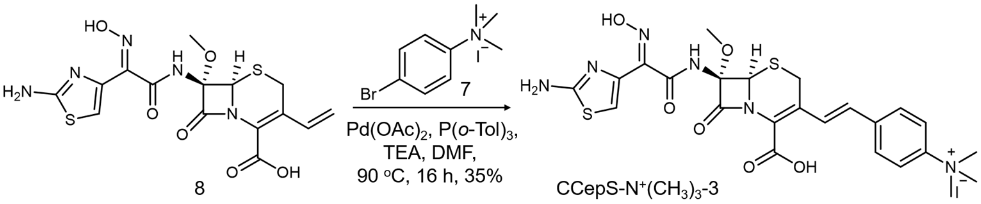

To a 150 mL boiling flask-3-neck was added compound **8** (2.13 g, 5 mmol), compound **7** (5.11 g, 15 mmol), $Pd(OAc)_2$ (673.53 mg, 3 mmol), $P(o\text{-}Tol)_3$ (913.11 mg, 3 mmol), triethylamine (3 mL) and anhydrous N, N-dimethylformamide (50 mL) under Ar atmosphere. The resulting reaction mixture was stirred at 90 °C for 16 h. When TLC indicated that the reaction was completed, the reaction solution was diluted with ethyl acetate (30 mL) and washed with 0.2 M HCl (30 mL), $NaHCO_3$ saturated aqueous solution (30 mL) and brine (50 mL) sequentially. The organic layer was dried over Mg_2SO_4 and concentrated. The crude product was purified by flash column chromatography with dichloromethane and methanol (DCM: MeOH = 10: 1) as eluent to afford **CCepS-N$^+$(CH$_3$)$_3$-3** (0.98 g, 29%) as a tan solid. ^{1}H NMR (400 MHz, DMSO) δ 13.56 (s, 1H), 11.37 (s, 1H), 9.51 (d, J = 8 Hz, 1H), 7.37 (d, J = 8 Hz, 2H), 7.10 (s, 2H), 7.00 (d, J = 16 Hz, 2H), 6.87 (d, J = 8 Hz, 2H), 6.76 (s, 1H), 6.69 (d, J = 16 Hz, 1H), 5.23 (d, J = 4 Hz, 1H), 3.94 (d, J = 16 Hz, 1H), 3.79 (s, 9H), 3.65 (d, J = 16 Hz, 1H), 3.55-3.46 (m, 3H). ^{13}C NMR (101 MHz, DMSO) δ 168.72, 164.47, 163.74, 148.99, 146.33, 143.68, 132.86, 132.47, 127.21, 125.97, 124.71, 119.34, 117.37, 107.26, 59.22, 58.35, 57.30, 52.03, 23.69. HRMS (ESI) m/z calcd for $C_{24}H_{27}N_6O_6S_2^+$ [M-I]$^+$ 559.1428, found 559.1455.

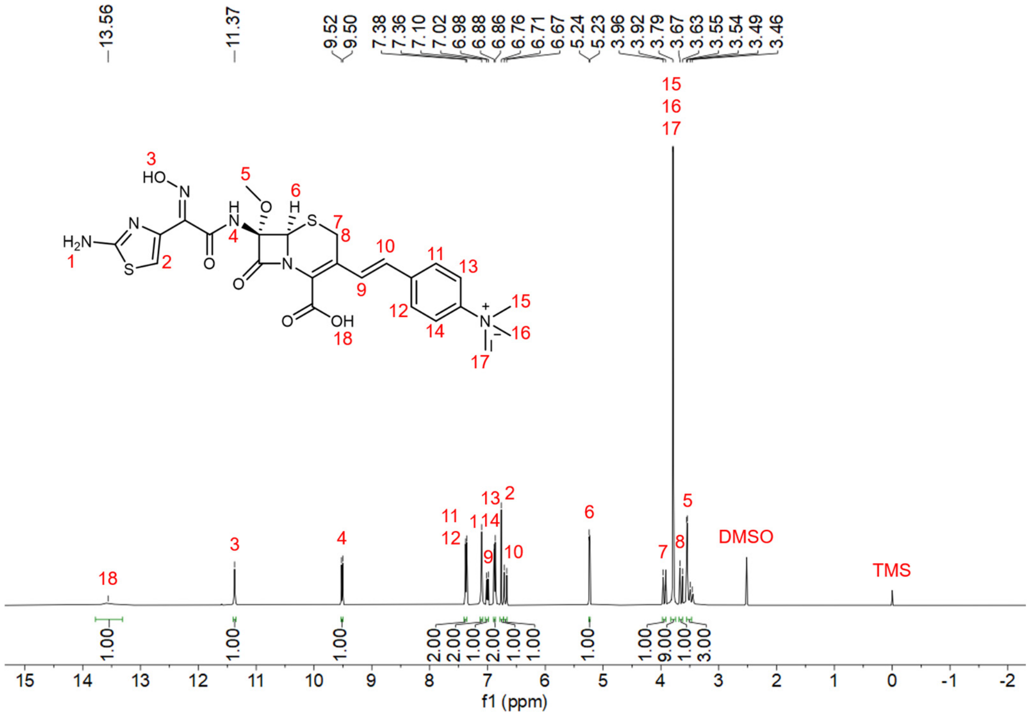

Supplementary Fig. 25 | ^{1}H NMR spectrum of CCepS-N$^+$(CH$_3$)$_3$-3 in DMSO.

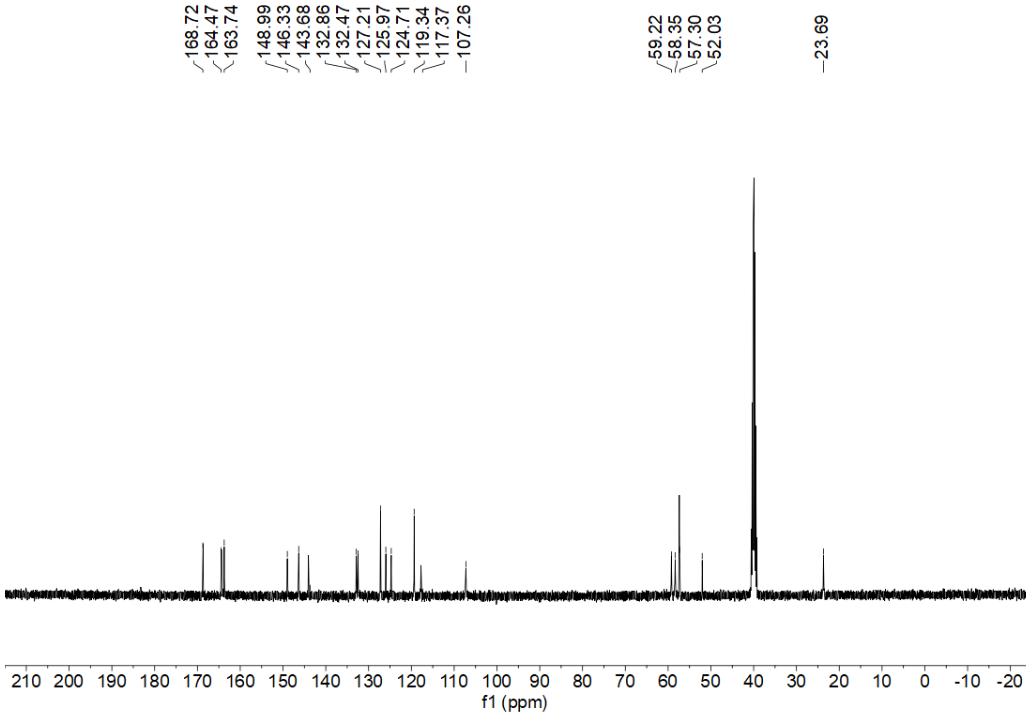

Supplementary Fig. 26 | ^{13}C NMR spectrum of CCepS-$N^+(CH_3)_3$-3 in DMSO.

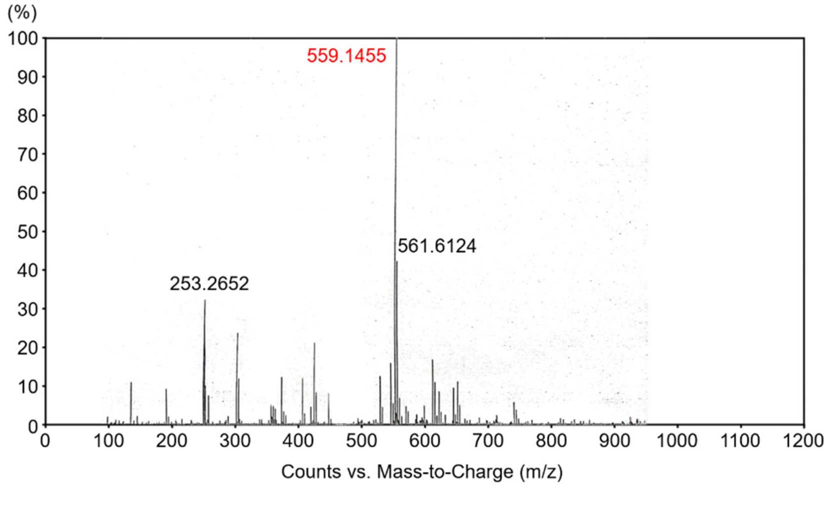

Supplementary Fig. 27 | HRMS spectrum (ESI) of CCepS-$N^+(CH_3)_3$-3.

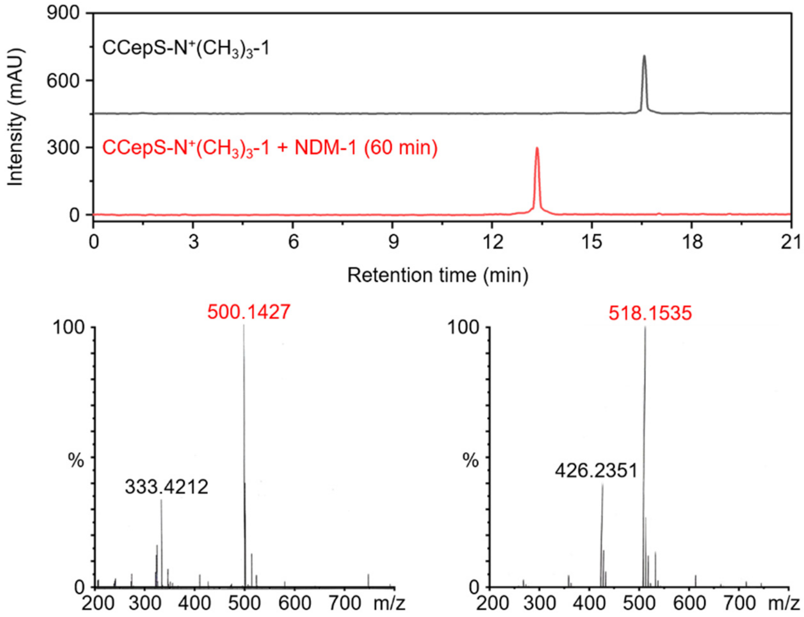

Supplementary Fig. 28 | HPLC-MS of CCepS-N$^+$(CH$_3$)$_3$-1 (10 μM, pH 7.4) before and after incubation with NDM-1 (3 nM) for 60 min.

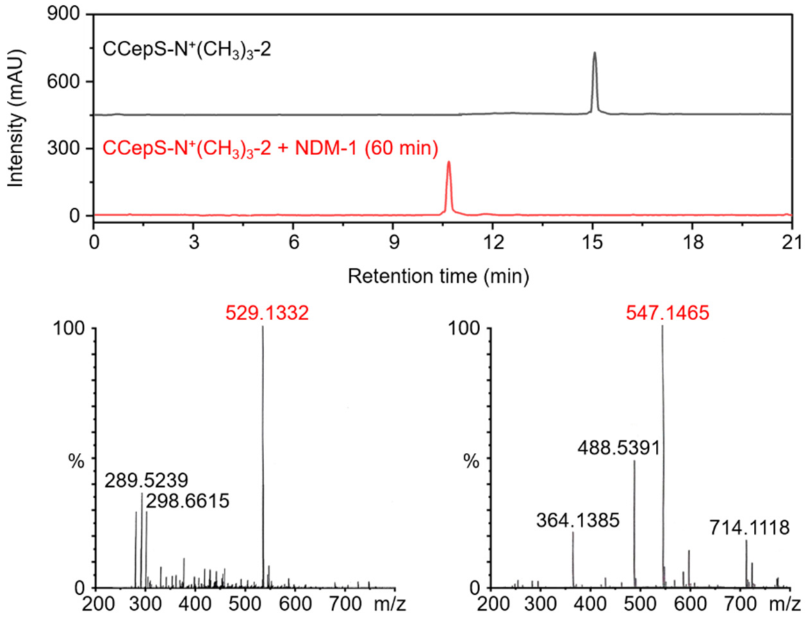

Supplementary Fig. 29 | HPLC-MS of CCepS-N$^+$(CH$_3$)$_3$-2 (10 μM, pH 7.4) before and after incubation with NDM-1 (3 nM) for 60 min.

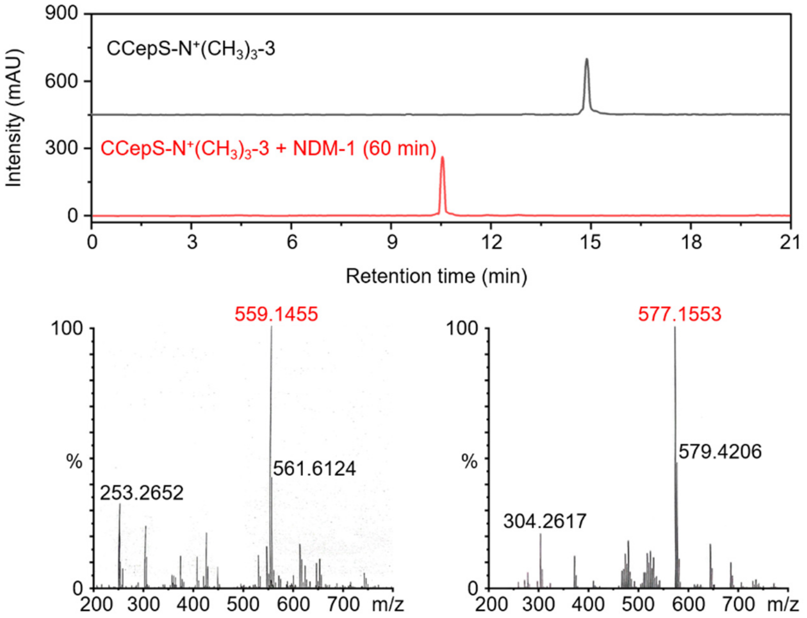

Supplementary Fig. 30 | HPLC-MS of CCepS-$N^+(CH_3)_3$-3 (10 µM, pH 7.4) before and after incubation with NDM-1 (3 nM) for 60 min.

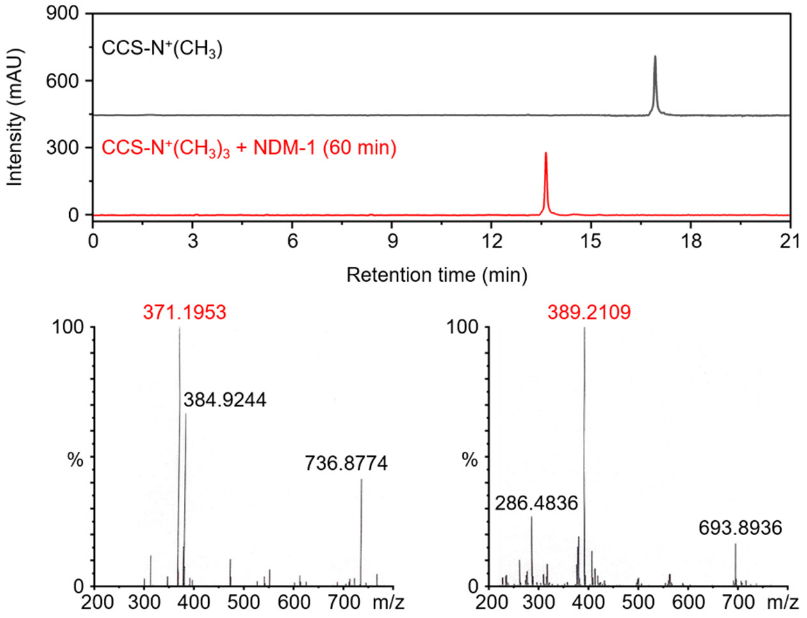

Supplementary Fig. 31 | HPLC-MS of CCS-$N^+(CH_3)_3$ (10 µM, pH 7.4) before and after incubation with NDM-1 (3 nM) for 60 min.

Supplementary Table 1 | Michaelis-Menten kinetics and LODs of **CCepS-N$^+$(CH$_3$)$_3$-1** for *β*-lactamases

***β*-lactamase**	**3S/k (pM)**	**k_{cat} (s^{-1})**	**K_m (μM)**	**k_{cat}/K_m (M^{-1} s^{-1})**
**NDM-1**	10.04	22.68 ± 0.55	1.96 ± 0.13	1.16×10^7
**IMP-4**	9.95	22.65 ± 0.26	1.88 ± 0.02	1.20×10^7
**KPC-3**	10.29	22.59 ± 0.37	1.79 ± 0.08	1.26×10^7
**VIM-27**	9.81	22.39 ± 0.31	1.78 ± 0.05	1.26×10^7
**OXA-48**	9.96	22.53 ± 0.17	1.80 ± 0.01	1.25×10^7
**MOX-1**	10.45	22.78 ± 0.29	1.81 ± 0.05	1.26×10^7
**CYM-4**	10.47	22.61 ± 0.65	1.79 ± 0.07	1.26×10^7
**TEM-7**	11.14	16.61 ± 0.23	1.75 ± 0.11	9.49×10^6
**SHV-5**	11.81	17.28 ± 0.52	2.04 ± 0.11	8.47×10^6
**CTX-M-3**	9.59	19.95 ± 0.72	1.81 ± 0.08	1.10×10^7
**TEM-1**	20.25	10.44 ± 0.45	1.36 ± 0.17	7.68×10^6

Supplementary Table 2 | Michaelis-Menten kinetics and LODs of CCepS-N$^+$(CH$_3$)$_3$-2 for *β*-lactamases

***β*-lactamase**	**3S/k (pM)**	**k_{cat} (s^{-1})**	**K_m (μM)**	**k_{cat}/K_m (M^{-1} s^{-1})**
**NDM-1**	8.27	22.61 ± 0.62	1.81 ± 0.19	1.25×10^7
**IMP-4**	8.26	22.78 ± 0.43	1.79 ± 0.04	1.27×10^7
**KPC-3**	8.32	22.65 ± 0.16	1.88 ± 0.09	1.21×10^7
**VIM-27**	7.96	22.45 ± 0.43	1.78 ± 0.11	1.26×10^7
**OXA-48**	7.73	22.43 ± 0.22	1.88 ± 0.07	1.20×10^7
**MOX-1**	9.34	22.56 ± 0.38	1.79 ± 0.02	1.26×10^7
**CYM-4**	9.07	22.84 ± 0.29	1.81 ± 0.04	1.26×10^7
**TEM-7**	13.61	11.17 ± 0.54	1.24 ± 0.06	9.01×10^6
**SHV-5**	14.86	10.64 ± 0.37	1.53 ± 0.09	6.95×10^6
**CTX-M-3**	8.43	20.11 ± 0.62	1.79 ± 0.05	1.12×10^7

Supplementary Table 3 │ Michaelis-Menten kinetics and LODs of CCepS-$N^+(CH_3)_3$-3 for *β*-lactamases

***β*-lactamase**	**3S/k (pM)**	**k_{cat} (s^{-1})**	**K_m (μM)**	**k_{cat}/K_m (M^{-1} s^{-1})**
**NDM-1**	9.71	21.99 ± 0.13	1.88 ± 0.15	1.17×10^7
**IMP-4**	10.56	22.75 ± 0.43	1.78 ± 0.08	1.28×10^7
**KPC-3**	9.81	22.24 ± 0.56	1.81 ± 0.04	1.23×10^7
**VIM-27**	9.59	22.45 ± 0.45	1.89 ± 0.07	1.19×10^7
**OXA-48**	10.01	22.43 ± 0.19	1.79 ± 0.12	1.25×10^7
**MOX-1**	9.76	22.27 ± 0.72	1.81 ± 0.02	1.23×10^7
**CYM-4**	10.61	22.32 ± 0.26	1.78 ± 0.08	1.25×10^7

Supplementary Table 4 │ Michaelis-Menten kinetics and LODs of CCS-$N^+(CH_3)_3$ for *β*-lactamases

***β*-lactamase**	**3S/k (pM)**	**k_{cat} (s^{-1})**	**K_m (μM)**	**k_{cat}/K_m (M^{-1} s^{-1})**
**NDM-1**	6.81	24.59 ± 1.18	1.16 ± 0.17	2.02×10^7
**IMP-4**	5.53	26.35 ± 1.06	1.37 ± 0.28	1.92×10^7
**KPC-3**	3.16	13.26 ± 0.39	0.71 ± 0.11	1.87×10^7
**VIM-27**	8.17	9.42 ± 0.06	1.21 ± 0.07	7.79×10^6
**OXA-48**	29.99	1.26 ± 0.03	0.75 ± 0.08	1.68×10^6

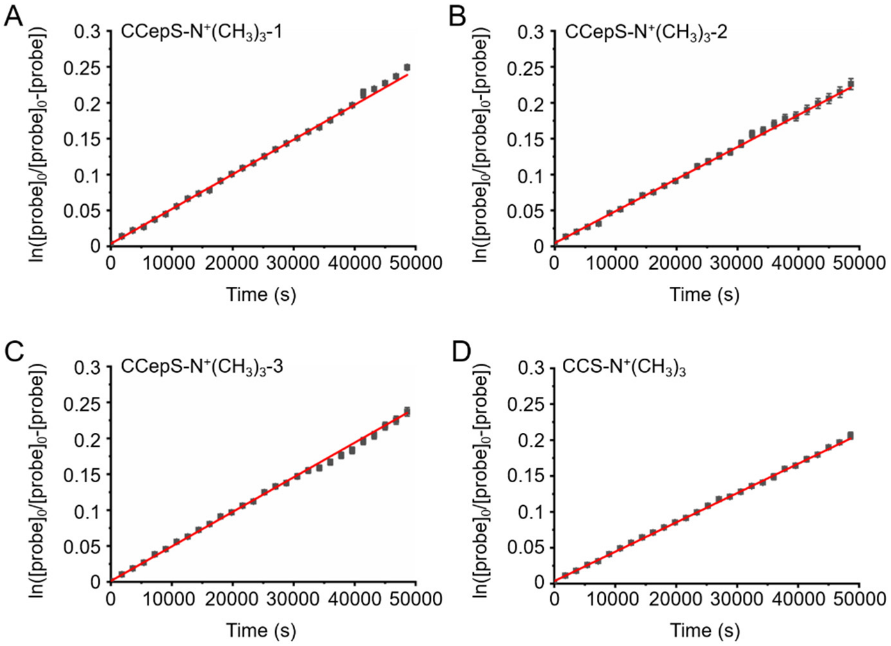

Supplementary Fig. 32 | First-order kinetics plots of the background hydrolysis of the probes including (A) CCepS-$N^+(CH_3)_3$-1, (B) CCepS-$N^+(CH_3)_3$-2, (C) CCepS-$N^+(CH_3)_3$-3, and (D) CCS-$N^+(CH_3)_3$. $[Probe]_0$ represents the initial probe concentration; [Probe] represents the probe concentration at a certain time point. Data represent mean ± s.d., n = 3, three technical replicates.

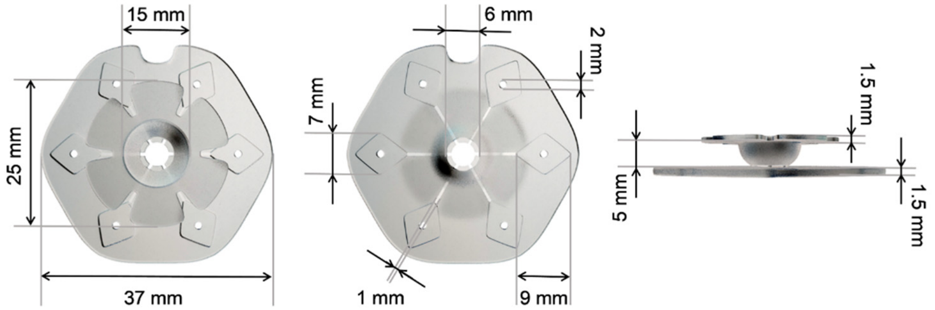

Supplementary Fig. 33 | The size information of the BSV sensor. A 2 mm diameter sized hole in the bottom of the chambers is linked to the central sampling hole by a channel (6 mm length × 1 mm width) , where the sample can be syphoned into the chambers in a few seconds.

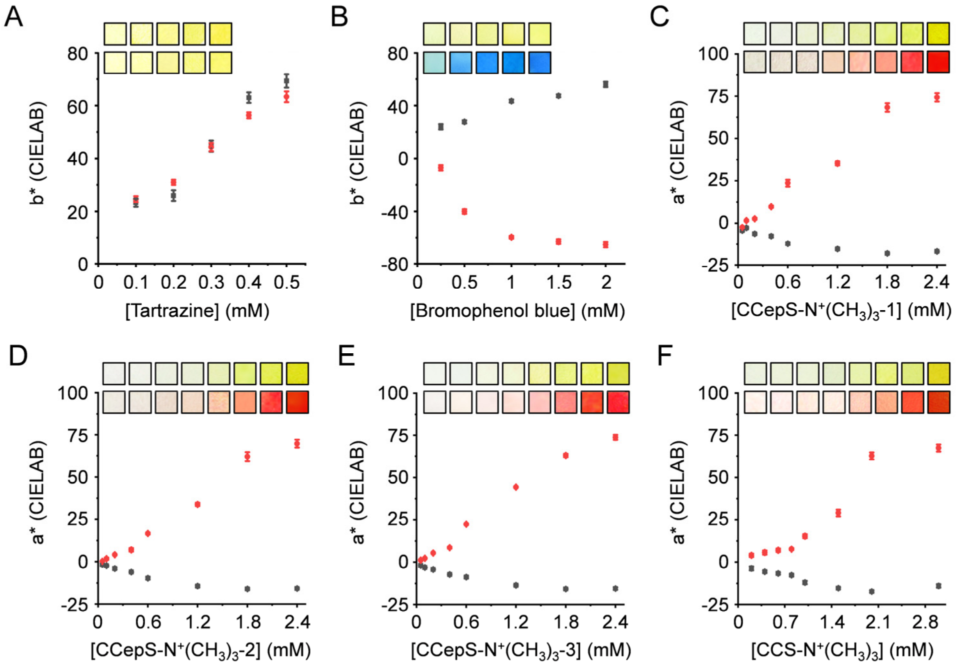

Supplementary Fig. 34 | Optimization of the chromogenic molecules loading in the BSV sensor. Color response and corresponding b* values of the papers loading with different concentrations of (A) tartrazine, (B) bromophenol blue, (C) **CCepS-N$^+$(CH$_3$)$_3$-1**, (D) **CCepS-N$^+$(CH$_3$)$_3$-2**, (E) **CCepS-N$^+$(CH$_3$)$_3$-3**, and (F) **CCS-N$^+$(CH$_3$)$_3$** before and after hydrolysis by carbapenemases (NDM-1, 3 nM, 10 μL). Data represent mean ± s.d., *n* = 3, three technical replicates.

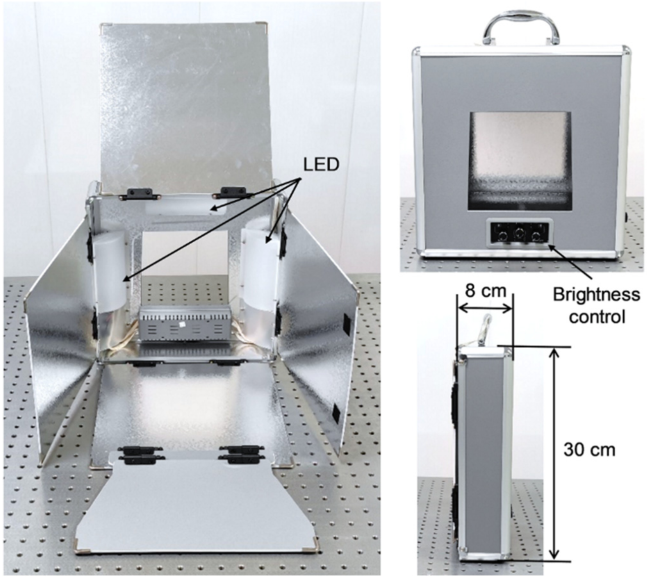

Supplementary Fig. 35 | Portable shadowless lamp in a hand-carrying case.

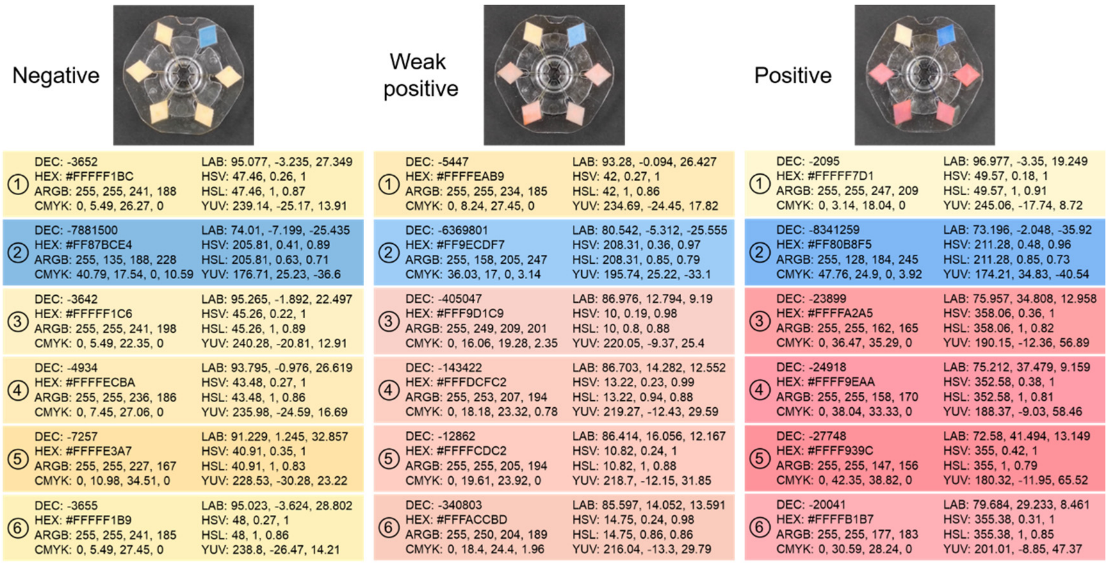

Supplementary Fig. 36 | Optimization of the color quantification mode. The intelligent smartphone app color recognizer is capable of converting image colors into 8 digital formats, including DEC, HEX, ARGB, CMYK, Lab, HSV, HSL, and YUV. Based on our experimental results, the Lab color mode is the optimal choice. This is because the Lab mode expresses color as 3 values: L* for the lightness from black to white, a* from green to red, and b* from blue to yellow.

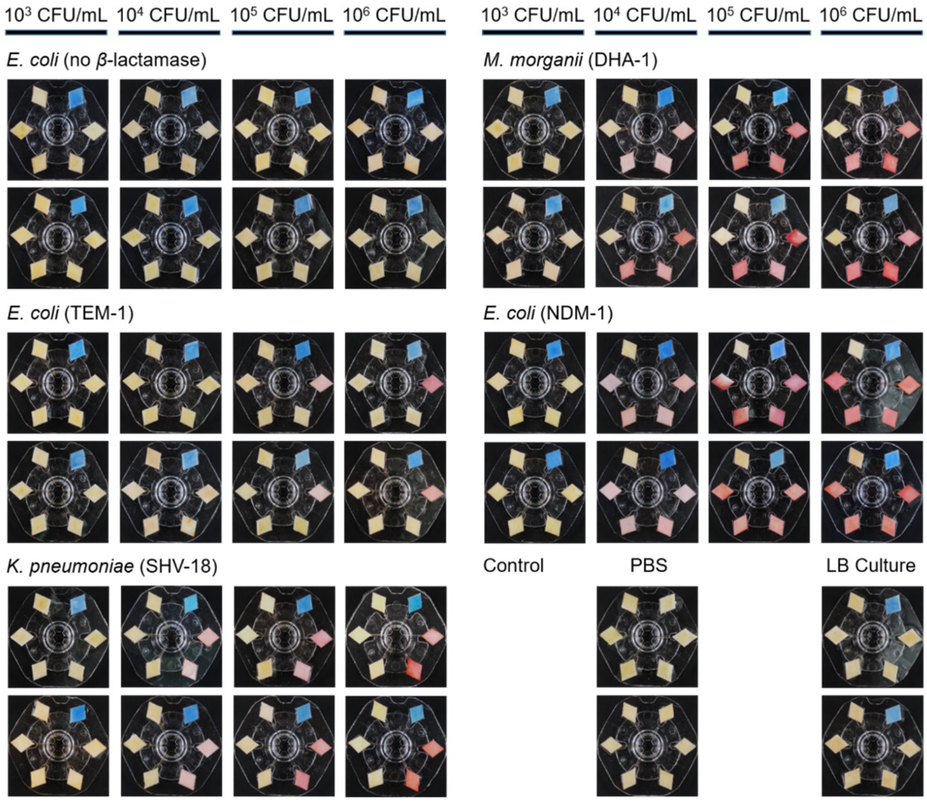

Supplementary Fig. 37 | Using the BSV sensor to classify the antibiotic resistance levels of the clinically isolated bacteria (another two replicates)**.**

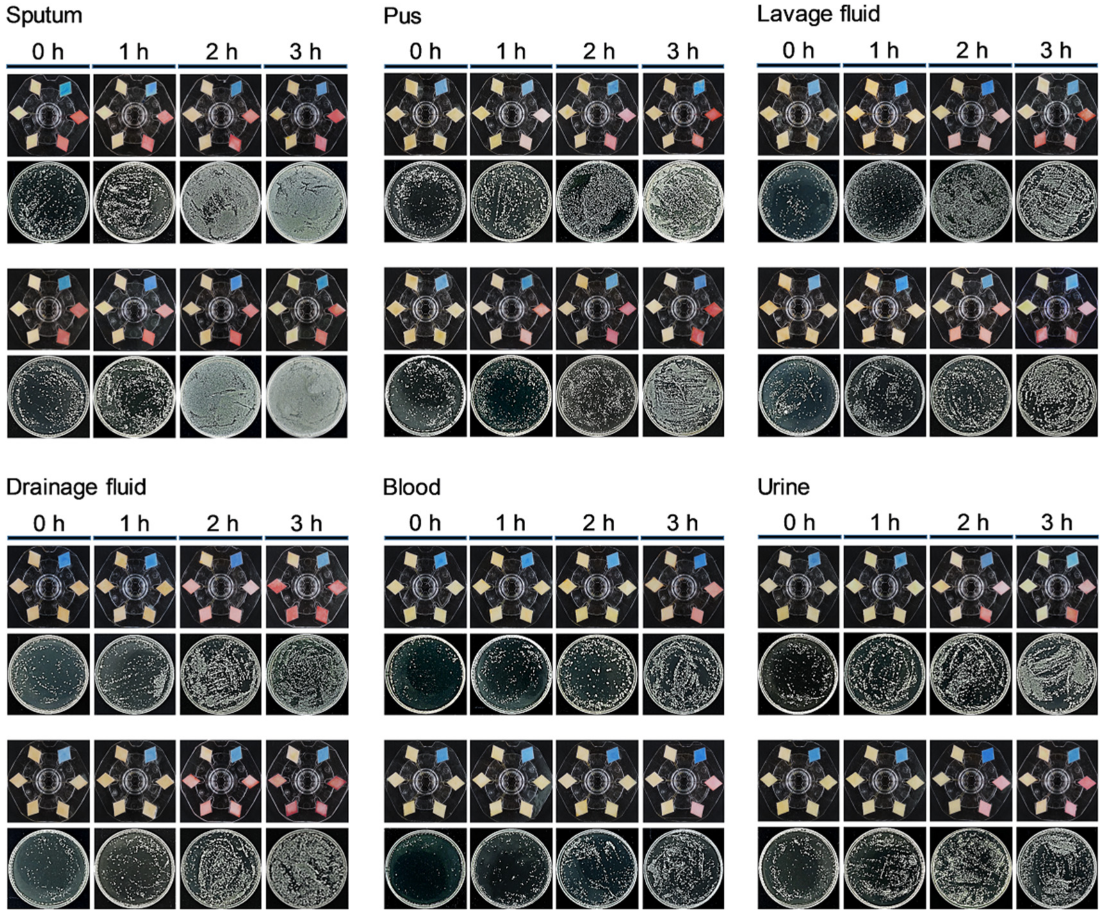

Supplementary Fig. 38 | Using the BSV sensor to classify the antibiotic resistance of diverse body fluid samples (another two replicates)**.**

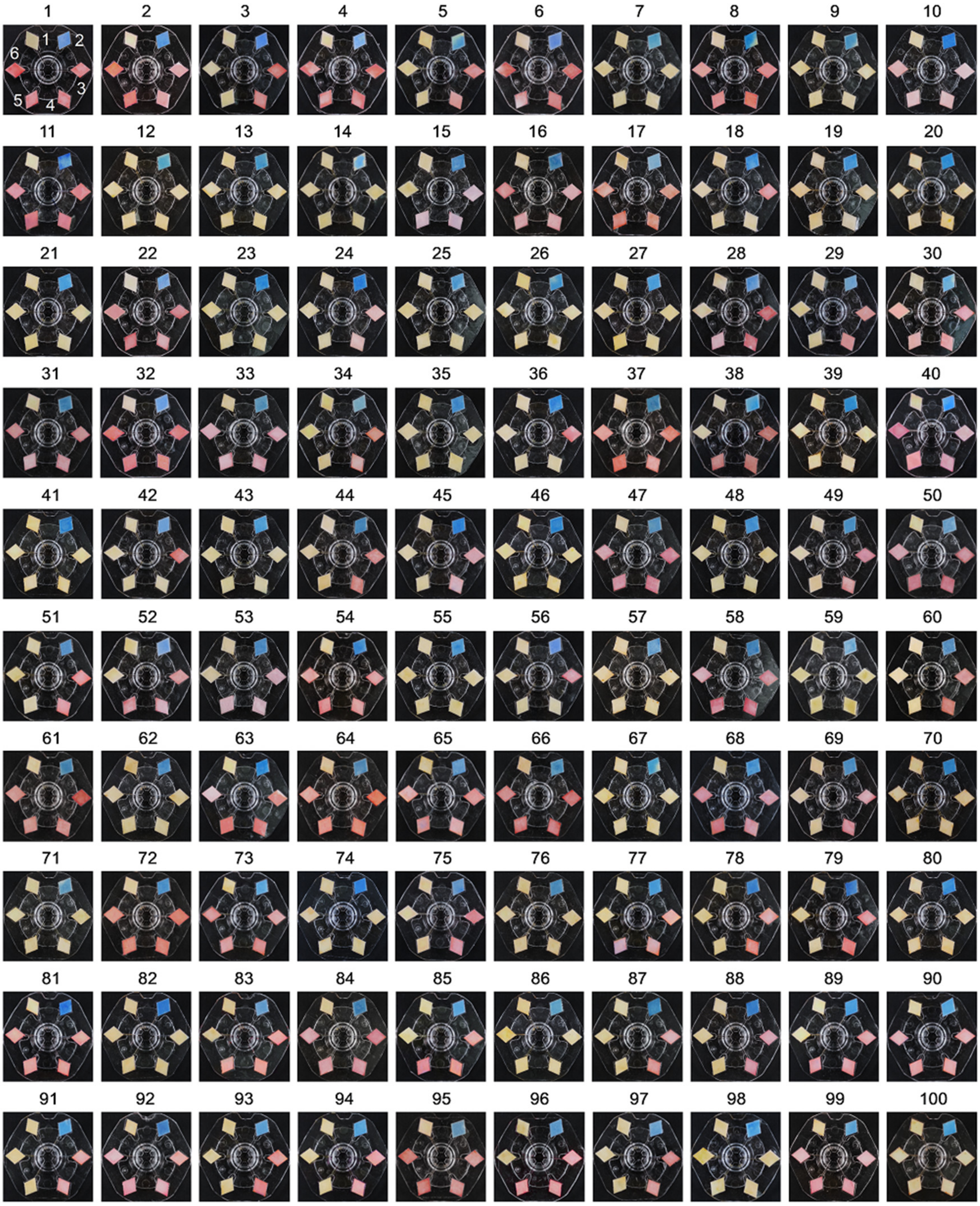

Supplementary Fig. 39 | Visual detection of antibiotic resistance in 100 clinical samples using the BSV sensor.

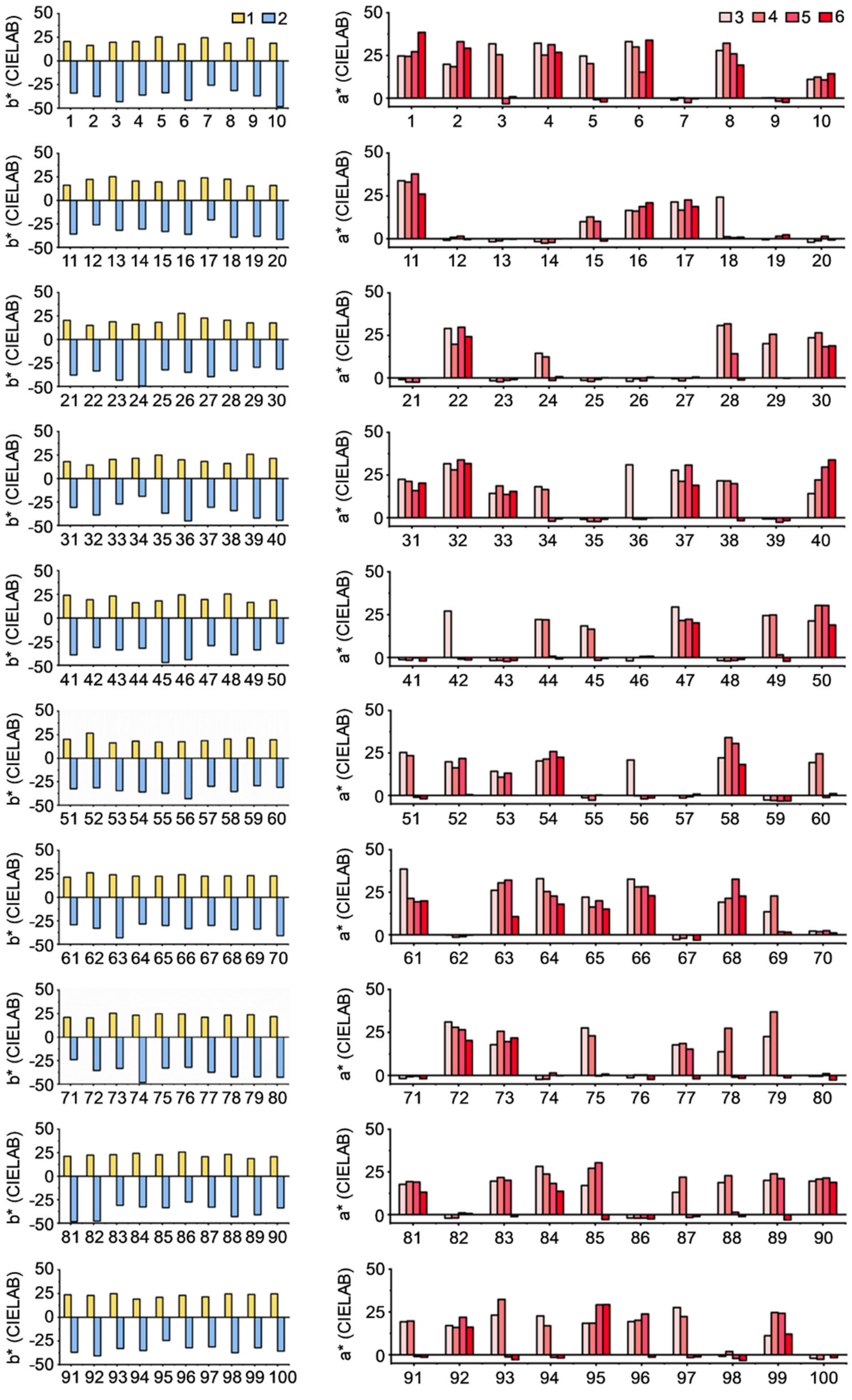

Supplementary Fig. 40 | Quantification of the results in Fig. S39 via the Lab mode of the smartphone app.

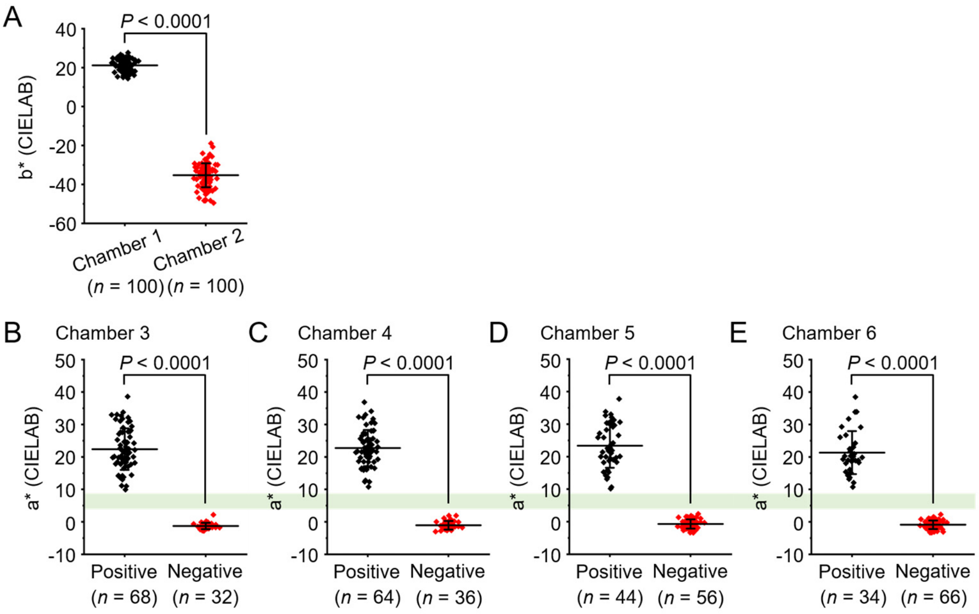

Supplementary Fig. 41 | Statistics of the a* and b* values shown in Supplementary Fig. 40. (A) The b* values of chambers 1 and 2. The a* values of (B) chamber 3, (C) chamber 4, (D) chamber 5, and (E) chamber 6. Paired two-tailed Student's *t* test, $P < 0.0001$. Error bars: mean ± s.d.

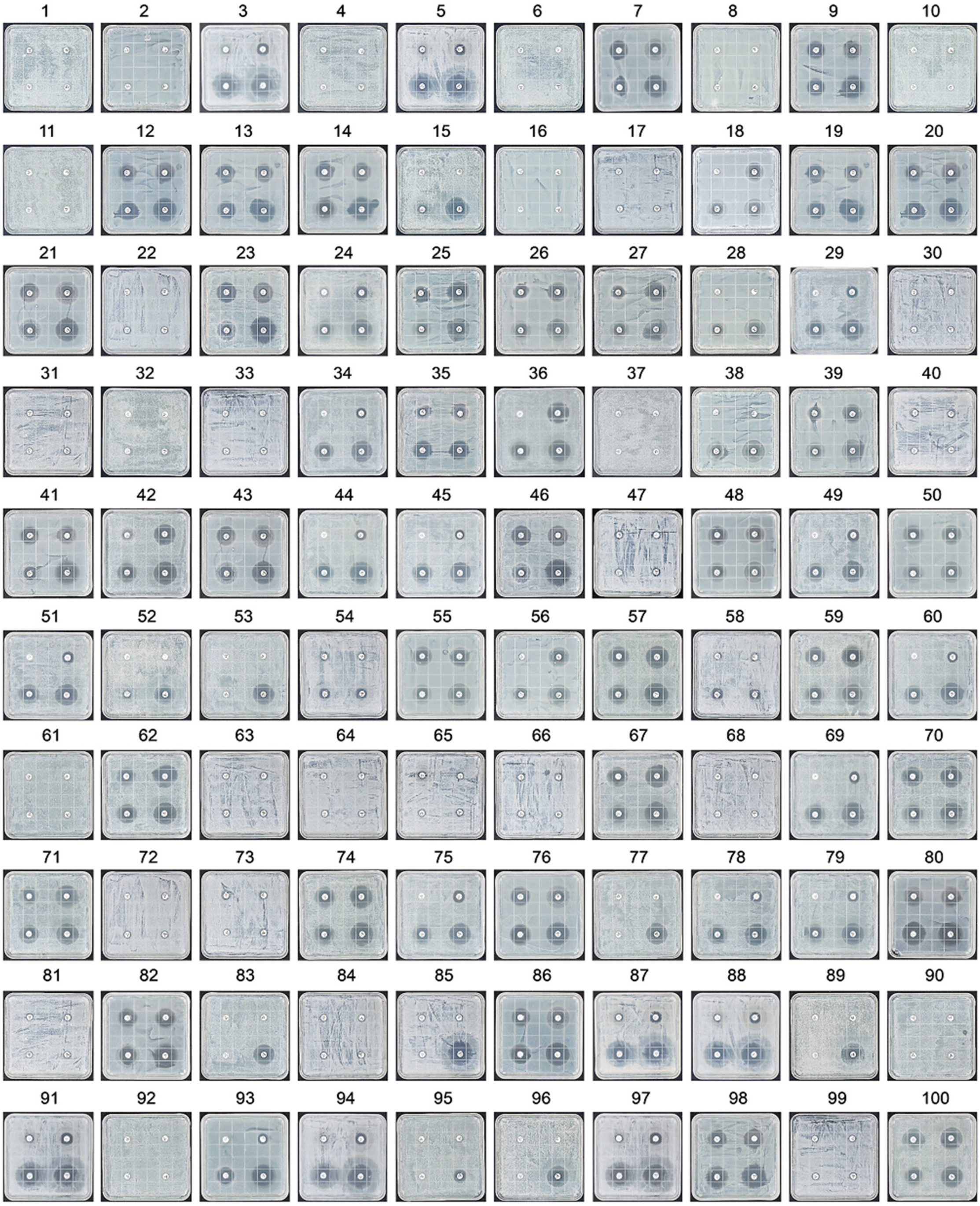

Supplementary Fig. 42 | Kirby-Bauer paper dispersion results for the 100 clinical samples.

Supplementary Table 5 | The diameters of the bacteriostatic zones in Supplementary Fig. 42

ID	Zone of inhibition (mm)	ID	Zone of inhibition (mm)	ID	Zone of inhibition (mm)	ID	Zone of inhibition (mm)
**1**	8, 8, 8, 8	**2**	8, 8, 8, 8	**3**	8, 17, 34, 37	**4**	8, 8, 8, 8
**5**	8, 17, 33, 36	**6**	8, 8, 8, 8	**7**	30, 33, 28, 36	**8**	8, 8, 8, 8
**9**	29, 29, 25, 35	**10**	8, 8, 8, 8	**11**	8, 8, 8, 8	**12**	27, 32, 32, 35
**13**	25, 26, 26, 34	**14**	31, 32, 33, 37	**15**	8, 8, 8, 35	**16**	8, 8, 8, 8
**17**	8, 8, 8, 8	**18**	8, 28, 28, 32	**19**	27, 27, 27, 37	**20**	28, 31, 33, 34
**21**	32, 36, 35, 35	**22**	8, 8, 8, 8	**23**	33, 30, 29, 38	**24**	8, 17, 24, 34
**25**	26, 36, 25, 34	**26**	24, 33, 29, 35	**27**	23, 35, 32, 35	**28**	8, 8, 15, 33
**29**	8, 17, 31, 33	**30**	8, 8, 8, 8	**31**	8, 8, 8, 8	**32**	8, 8, 8, 8
**33**	8, 8, 8, 8	**34**	8, 14, 32, 34	**35**	21, 25, 29, 29	**36**	8, 31, 27, 33
**37**	8, 8, 8, 8	**38**	8, 8, 12, 34	**39**	23, 33, 33, 35	**40**	8, 10, 8, 9
**41**	29, 29, 19, 35	**42**	10, 33, 28, 34	**43**	31, 34, 33, 34	**44**	8, 17, 25, 34
**45**	8, 14, 32, 34	**46**	35, 34, 30, 37	**47**	8, 8, 8, 8	**48**	34, 32, 32, 34
**49**	8, 8, 32, 34	**50**	32, 33, 32, 33	**51**	8, 17, 31, 34	**52**	8, 8, 10, 33
**53**	8, 8, 8, 34	**54**	10, 10, 15, 15	**55**	31, 31, 28, 34	**56**	8, 32, 28, 34
**57**	28, 34, 29, 35	**58**	8, 8, 13, 12	**59**	29, 31, 23, 29	**60**	8, 15, 27, 35
**61**	8, 8, 8, 8	**62**	26, 35, 28, 34	**63**	8, 10, 10, 10	**64**	8, 8, 8, 8
**65**	11, 10, 8, 10	**66**	8, 10, 10, 8	**67**	25, 33, 24, 34	**68**	8, 8, 8, 8
**69**	8, 16, 30, 34	**70**	28, 33, 25, 33	**71**	25, 33, 28, 34	**72**	8, 8, 8, 8
**73**	13, 11, 8, 11	**74**	28, 34, 28, 35	**75**	8, 16, 31, 34	**76**	33, 29, 29, 36
**77**	8, 8, 8, 33	**78**	8, 17, 32, 34	**79**	8, 15, 32, 34	**80**	32, 31, 34, 36
**81**	8, 8, 8, 8	**82**	30, 32, 29, 37	**83**	8, 8, 8, 32	**84**	8, 8, 8, 8
**85**	8, 8, 8, 35	**86**	29, 34, 31, 34	**87**	8, 15, 35, 37	**88**	8, 13, 29, 35
**89**	8, 8, 8, 34	**90**	8, 8, 8, 8	**91**	10, 18, 33, 36	**92**	8, 8, 8, 8
**93**	8, 17, 28, 35	**94**	8, 16, 33, 38	**95**	8, 8, 8, 17	**96**	8, 8, 8, 32
**97**	9, 17, 33, 37	**98**	30, 31, 34, 36	**99**	8, 8, 8, 8	**100**	32, 34, 25, 33

Supplementary Table 6 | The sequences of the designed primers for diverse carbapenemase genes

Target gene	Primer (5′–3′)	ACCESSION CODE [hyperlink]
**All *bla*$_{NDM}$ alleles**	F: GATTGCGACTTATGCCAATG<br>R: TCGATCCCAACGGTGATATT	GenBank accession CP034323.1 [https://www.ncbi.nlm.nih.gov/nuccore/CP034323.1]
**All *bla*$_{IMP}$ alleles**	F: GAGTGGCTTAATTCTCRATC<br>R: AACTAYCCAATAYRTAAC	GenBank accession JX648311.1 [https://www.ncbi.nlm.nih.gov/nuccore/JX648311.1]
**All *bla*$_{KPC}$ alleles**	F: CAGCTCATTCAAGGGCTTTC<br>R: GGCGGCGTTATCACTGTATT	GenBank accession JN974188.1 [https://www.ncbi.nlm.nih.gov/nuccore/JN974188.1]
**All *bla*$_{VIM}$ alleles**	F: GTTTGGTCGCATATCGCAAC<br>R: AATGCGCAGCACCAGGATAG	GenBank accession AY648125.1 [https://www.ncbi.nlm.nih.gov/nuccore/AY648125.1]
**All *bla*$_{GES}$ alleles**	F: ATGCGCTTCATTCACGCAC<br>R: CTATTTGTCCGTGCTCAGG	GenBank accession AF156486.1 [https://www.ncbi.nlm.nih.gov/nuccore/AF156486.1]
**All *bla*$_{SME}$ alleles**	F: AACGGCTTCATTTTTGTTTAG<br>R: GCTTCCGCAATAGTTTTATCA	GenBank accession U60295.1 [https://www.ncbi.nlm.nih.gov/nuccore/U60295.1]
***bla*$_{OXA-48}$ and related alleles**	F: TGCGCTTTCCTTTCTGTCAAG<br>R: CGCACTTTCTCCGCAGTTT	GenBank accession CP034202.2 [https://www.ncbi.nlm.nih.gov/nuccore/CP034202.2]

Supplementary Table 7 | The sequences of the designed primers for diverse AmpC genes

Target gene	Primer (5′–3′)	ACCESSION CODE [hyperlink]
**All *bla*DHA alleles**	F: AACTTTCACAGGTGTGCTG<br>R: ACTCTTTCGGTATTCGGGT	GenBank accession Y16410.1 [https://www.ncbi.nlm.nih.gov/nuccore/Y16410.1]
**All *bla*CMY alleles**	F: ATGATGAAAAAATCGTTATGCT<br>R: TTATTGCAGCTTTTCAAGAATGCG	GenBank accession X92508.1 [https://www.ncbi.nlm.nih.gov/nuccore/X92508.1]
**All *bla*FOX alleles**	F: ACGGCTATTCGAAGGAAGA<br>R: TCGATGGGATAGTTGCGAT	GenBank accession X77455.1 [https://www.ncbi.nlm.nih.gov/nuccore/X77455.1]
**All *bla*MOX alleles**	F: TGCAATTCCCCGASGAGGT<br>R: GTTGCGRTTGGCCAGCAT	GenBank accession AJ276453.1 [https://www.ncbi.nlm.nih.gov/nuccore/AJ276453.1]
**All *bla*MIR alleles**	F: CAAAATCCCTAAGCTGTGCC<br>R: CGGCTGCCAGTTTTGATAAA	GenBank accession M37839.1 [https://www.ncbi.nlm.nih.gov/nuccore/M37839.1]
**All *bla*ACC alleles**	F: CCCATCGCGTTTATTCCAA<br>R: GAGCAAATTCGGCAGAGAA	GenBank accession AY856832.1 [https://www.ncbi.nlm.nih.gov/nuccore/AY856832.1]
**All *bla*CIT alleles**	F: TGGCCAGAACTGACAGGCAAA<br>R: TTTCTCCTGAACGTGGCTGGC	GenBank accession X78117.1 [https://www.ncbi.nlm.nih.gov/nuccore/X78117.1]
**All *bla*EBC alleles**	F: TCGGTAAAGCCGATGTTGCGG<br>R: CTTCCACTGCGGCTGCCAGTT	GenBank accession M37839.2 [https://www.ncbi.nlm.nih.gov/nuccore/M37839.2]

Supplementary Table 8 | The sequences of the designed primers for diverse ESBL genes

Target gene	Primer (5′–3′)	ACCESSION CODE
**All *bla*TEM alleles**	F: GCGGAACCCCTATTTG<br>R: ACCAATGCTTAATCAGTGAG	GenBank accession DQ679961.1 [https://www.ncbi.nlm.nih.gov/nuccore/DQ679961.1]
**All *bla*SHV alleles**	F: TTATCTCCCTGTTAGCCACC<br>R: GATITGCTGATTTCGCTCGG	GenBank accession AY293070.1 [https://www.ncbi.nlm.nih.gov/nuccore/AY293070.1]
**All *bla*CTX-M**	F: CGATGTGCAGTACCAGTAA<br>R: TTAGTGACCAGAATCAGCGG	GenBank accession AF550415.1 [https://www.ncbi.nlm.nih.gov/nuccore/AF550415.1]
**All *bla*VEB alleles**	F: CGACTTCCATTTCCCGATGC<br>R: GGACTCTGCAACAAATACGC	GenBank accession KU356480.1 [https://www.ncbi.nlm.nih.gov/nuccore/KU356480.1]
**All *bla*OXA alleles**	F: AGCCGTTAAAATTAAGCCC<br>R: CTTGATTGAAGGGTTGGGCG	GenBank accession J02967.2 [https://www.ncbi.nlm.nih.gov/nuccore/J02967.2]

Supplementary Table 9 | The sequences of the designed primers for diverse BSBL genes

Target gene	Primer (5′–3′)	ACCESSION CODE [hyperlink]
***bla*$_{TEM-1}$ and *bla*$_{TEM-2}$ alleles**	F: CATTTCCGTGTCGCCCTTATTC<br>R: CGTTCATCCATAGTTGCCTGAC	GenBank accession AY394610.1 [https://www.ncbi.nlm.nih.gov/nuccore/AY394610.1]
***bla*$_{SHV-1}$ alleles**	F: AGCCGCTTGAGCAAATTAAAC<br>R: ATCCCGCAGATAAATCACCAC	GenBank accession AF124984.1 [https://www.ncbi.nlm.nih.gov/nuccore/AF124984.1]

Supplementary Table 10 | RT-PCR reaction system

Reagent	50 µL Reaction	Final concentration
2× SGExcel FastSYBR	25 µL	-
Forward primer, 5 µM	3 µL	0.3 µM
Reverse primer, 5 µM	3 µL	0.3 µM
Sample	5 µL	-
RNase-Free ddH_2O	14 µL	-

Supplementary Table 11 | RT-PCR response procedures

Procedure	Temperature/°C	Time/s
**Predenaturation**	94	300
**Denaturation**	94	30
**Annealing**	56	45
**Extension**	72	30
	94	10
**Melting analysis**	72	60
	94	1
