# Supplementary material for: Rapid and visual identification of β-lactamase subtypes for precision antibiotic therapy
Source: Nat Commun. 2024 Jan 24;15:719. doi: 10.1038/s41467-024-44984-y (PMC10808423; doi:10.1038/s41467-024-44984-y)
Supplement: Supplementary file 3 — Description of Additional Supplementary Files [file 41467_2024_44984_MOESM3_ESM.pdf]

Description of Additional Supplementary Fles Document

File name: Supplementary Data 1

Description: Contains ID, sex, age, infection site, sample, time to result, diagnostic results, initial medication, and targeted medication of the patients
