# Supplementary material for: Rapid and visual identification of β-lactamase subtypes for precision antibiotic therapy
Source: Nat Commun. 2024 Jan 24;15:719. doi: 10.1038/s41467-024-44984-y (PMC10808423; doi:10.1038/s41467-024-44984-y)
Supplement: Supplementary file 5 — Reporting Summary [file 41467_2024_44984_MOESM5_ESM.pdf]

nature portfolio

Corresponding author(s): Dingbin Liu

Last updated by author(s): Dec 20, 2023

Reporting Summary

Nature Portfolio wishes to improve the reproducibility of the work that we publish. This form provides structure for consistency and transparency in reporting. For further information on Nature Portfolio policies, see our Editorial Policies and the Editorial Policy Checklist.

Statistics

For all statistical analyses, confirm that the following items are present in the figure legend, table legend, main text, or Methods section.

n/a	Confirmed	
☐	☒	The exact sample size (*n*) for each experimental group/condition, given as a discrete number and unit of measurement
☐	☒	A statement on whether measurements were taken from distinct samples or whether the same sample was measured repeatedly
☐	☒	The statistical test(s) used AND whether they are one- or two-sided<br>*Only common tests should be described solely by name; describe more complex techniques in the Methods section.*
☒	☐	A description of all covariates tested
☒	☐	A description of any assumptions or corrections, such as tests of normality and adjustment for multiple comparisons
☐	☒	A full description of the statistical parameters including central tendency (e.g. means) or other basic estimates (e.g. regression coefficient) AND variation (e.g. standard deviation) or associated estimates of uncertainty (e.g. confidence intervals)
☐	☒	For null hypothesis testing, the test statistic (e.g. F, t, r) with confidence intervals, effect sizes, degrees of freedom and P value noted<br>*Give P values as exact values whenever suitable.*
☒	☐	For Bayesian analysis, information on the choice of priors and Markov chain Monte Carlo settings
☒	☐	For hierarchical and complex designs, identification of the appropriate level for tests and full reporting of outcomes
☒	☐	Estimates of effect sizes (e.g. Cohen's *d*, Pearson's *r*), indicating how they were calculated

Our web collection on statistics for biologists contains articles on many of the points above.

Software and code

Policy information about availability of computer code

Data collection	UV Probe Software v2.42, LightCycler 96 Software v1.1, Bruker Xenon Software v1.2, Prime Software v6.0, Colour Recognition App v1.5
Data analysis	Origin 2018 was used for data analyzing and plotting. MestReNova (Version 14.2.3-29241) was used for NMR data processing. Image J (Version 1.54f) was used for bacterial colony count. GraphPad Prism 9 for unpaired two-tailed Student's t-test with Welch's correction.

For manuscripts utilizing custom algorithms or software that are central to the research but not yet described in published literature, software must be made available to editors and reviewers. We strongly encourage code deposition in a community repository (e.g. GitHub). See the Nature Portfolio guidelines for submitting code & software for further information.

Data

Policy information about availability of data

All manuscripts must include a data availability statement. This statement should provide the following information, where applicable:

- Accession codes, unique identifiers, or web links for publicly available datasets
- A description of any restrictions on data availability
- For clinical datasets or third party data, please ensure that the statement adheres to our policy

All data needed to evaluate the conclusions in the paper are available in the main text or the Supplementary Information. Source data are provided with this paper. There is no restriction on data availability. No code was developed in this study.

Research involving human participants, their data, or biological material

Policy information about studies with human participants or human data. See also policy information about sex, gender (identity/presentation), and sexual orientation and race, ethnicity and racism.

Reporting on sex and gender	All cases were randomized into groups. There was no sex bias.
Reporting on race, ethnicity, or other socially relevant groupings	All of the cases were all Asian and indigenous to China.
Population characteristics	Information on sex, age, disease duration, and treatment can be found in file Supplementary Data 1.
Recruitment	All biological samples and case information were obtained from the Department of Critical Care Medicine, Tianjin First Central Hospital. The 100 patients involve various infectious diseases, including 68 cases of pulmonary infections, 14 cases of urinary tract infections, 7 cases of abdominal infections, 4 cases of liver abscesses, 2 cases of gallbladder abscesses, 1 case of neck abscess, 1 case of sinusitis, 1 case of pancreatitis, 1 case of pelvic inflammatory disease, and 1 case of leg inflammation. These clinical specimens include 56 sputum samples, 14 urine samples, 9 bronchoalveolar lavage fluids, 7 peritoneal drainage fluids, 4 hepatic drainage fluids, 2 gallbladder drainage fluids, 2 blood samples, 1 pleural effusion, 1 neck abscess fluid, 1 pelvic effusion, 1 sinus lavage fluid, 1 pancreatic drainage fluid, and 1 leg abscess fluid (Figure 5B).
Ethics oversight	The study was approved by the Nankai University Institutional Review Board (NKU IRB). All human samples were acquired and handled according to the protocols approved by the Scientific Ethical Committee of the First Central Hospital of Tianjin (Approval No. 2023DZX12). Informed consent was obtained and no compensation was provided for all research participants.

Note that full information on the approval of the study protocol must also be provided in the manuscript.

Field-specific reporting

Please select the one below that is the best fit for your research. If you are not sure, read the appropriate sections before making your selection.

☒ Life sciences ☐ Behavioural & social sciences ☐ Ecological, evolutionary & environmental sciences

For a reference copy of the document with all sections, see nature.com/documents/nr-reporting-summary-flat.pdf

Life sciences study design

All studies must disclose on these points even when the disclosure is negative.

Sample size	No sample size calculation was performed. Sample size was determined by the number of patients in intensive care unit, from the start of our program to the start of sensing (5th January 2023 - 21th January 2023), and where samples at the time points chosen for this study were available. The process of sample collection is uninterrupted until the 100th sample is collected. The 100 patients involve various infectious diseases, including 68 cases of pulmonary infections, 14 cases of urinary tract infections, 7 cases of abdominal infections, 4 cases of liver abscesses, 2 cases of gallbladder abscesses, 1 case of neck abscess, 1 case of sinusitis, 1 case of pancreatitis, 1 case of pelvic inflammatory disease, and 1 case of leg inflammation. These clinical specimens include 56 sputum samples, 14 urine samples, 9 bronchoalveolar lavage fluids, 7 peritoneal drainage fluids, 4 hepatic drainage fluids, 2 gallbladder drainage fluids, 2 blood samples, 1 pleural effusion, 1 neck abscess fluid, 1 pelvic effusion, 1 sinus lavage fluid, 1 pancreatic drainage fluid, and 1 leg abscess fluid (Figure 5B).
Data exclusions	No data was excluded from the analysis
Replication	Three independent experiments were performed for all studies of probe performance (Figure 2B-E). The detection accuracy of the chip results was validated by the Kirby-Bauer paper dispersion method (K-B method) for AST and RT-PCR verification (Figure 5A and Supplementary Figure 42).
Randomization	The order of samples were randomized for detection.
Blinding	Our study did not involve a blinded experiment. All data collected was quantifiable and blinding would not mitigate any subjective biases. Samples were measured by the same protocol, and were not allocated to any group.

Reporting for specific materials, systems and methods

We require information from authors about some types of materials, experimental systems and methods used in many studies. Here, indicate whether each material, system or method listed is relevant to your study. If you are not sure if a list item applies to your research, read the appropriate section before selecting a response.

Materials & experimental systems

n/a	Involved in the study
☒	☐ Antibodies
☒	☐ Eukaryotic cell lines
☒	☐ Palaeontology and archaeology
☒	☐ Animals and other organisms
☒	☐ Clinical data
☒	☐ Dual use research of concern
☒	☐ Plants

Methods

n/a	Involved in the study
☒	☐ ChIP-seq
☒	☐ Flow cytometry
☒	☐ MRI-based neuroimaging
